# Characterization of the Wood Mycobiome of *Vitis vinifera* in a Vineyard Affected by Esca. Spatial Distribution of Fungal Communities and Their Putative Relation With Leaf Symptoms

**DOI:** 10.3389/fpls.2019.00910

**Published:** 2019-07-12

**Authors:** Giovanni Del Frari, Alex Gobbi, Marie Rønne Aggerbeck, Helena Oliveira, Lars Hestbjerg Hansen, Ricardo Boavida Ferreira

**Affiliations:** ^1^LEAF – Linking Landscape, Environment, Agriculture and Food, Instituto Superior de Agronomia, University of Lisbon, Lisbon, Portugal; ^2^Environmental Microbial Genomics Group, Section for Environmental Microbiology and Biotechnology, Department of Environmental Science, Aarhus University, Roskilde, Denmark

**Keywords:** microbial ecology, *Vitis*, metabarcoding, mycobiome, esca disease, grapevine trunk diseases

## Abstract

Esca is a disease complex belonging to the grapevine trunk diseases cluster. It comprises five syndromes, three main fungal pathogenic agents and several symptoms, both internal (i.e., affecting woody tissue) and external (e.g., affecting leaves and bunches). The etiology and epidemiology of this disease complex remain, in part, unclear. Some of the points that are still under discussion concern the sudden rise in disease incidence, the simultaneous presence of multiple wood pathogens in affected grapevines, the causal agents and the discontinuity in time of leaf symptoms manifestation. The standard approach to the study of esca has been mostly through culture-dependent studies, yet, leaving many questions unanswered. In this study, we used Illumina^®^ next-generation amplicon sequencing to investigate the mycobiome of grapevines wood in a vineyard with history of esca. We characterized the wood mycobiome composition, investigated the spatial dynamics of the fungal communities in different areas of the stem and in canes, and assessed the putative link between mycobiome and leaf symptoms. An unprecedented diversity of fungi is presented (289 taxa), including five genera reported for the first time in association with grapevines wood (*Debaryomyces*, *Trematosphaeria*, *Biatriospora*, *Lopadostoma*, and *Malassezia*) and numerous hitherto unreported species. Esca-associated fungi *Phaeomoniella chlamydospora* and *Fomitiporia* sp. dominate the fungal community, and numerous other fungi associated with wood syndromes are also encountered (e.g., *Eutypa* spp., *Inonotus hispidus*). The spatial analysis revealed differences in diversity, evenness and taxa abundances, the unique presence of certain fungi in specific areas of the plants, and tissue specificity. Lastly, the mycobiome composition of the woody tissue in proximity to leaves manifesting ‘tiger stripes’ symptoms of esca, as well as in leaf-symptomatic canes, was highly similar to that of plants not exhibiting any leaf symptomatology. This observation supports the current understanding that leaf symptoms are not directly linked with the fungal communities in the wood. This work builds to the understanding of the microbial ecology of the grapevines wood, offering insights and a critical view on the current knowledge of the etiology of esca.

## Introduction

The phyllosphere, rhizosphere, and endosphere of grapevine (*Vitis vinifera* L.) are characterized by the presence of complex communities of microorganisms that constantly interact with one another and with the plant, affecting it positively, neutrally or negatively (Bruez et al., 2014; Pinto et al., 2014; Zarraonaindia et al., 2015). Until a decade ago, the approach to characterize the mycobiome – namely the fungal community present in/on an organism – of grapevines, focused on culture-dependent studies in which fungi were isolated *in vitro* and identified morphologically and/or molecularly (Morgan et al., 2017). This approach remains valid to this day, however, it presents several limitations, such as the impossibility of detecting uncultivable fungi, the bias of the cultivation conditions (e.g., growth medium, incubation parameters) and the difficulty of isolating species present in low abundances (Morgan et al., 2017). In recent years, technologies like next-generation sequencing (NGS) have improved in quality and reduced in cost, which, in combination with ever more efficient bioinformatics tools, have allowed the exploitation of this method in the study of the molecular ecology of environmental DNA (eDNA) samples. In particular, DNA metabarcoding approaches have taken the investigations of microbiomes to a new level, surpassing some of the limitations which characterize culture-dependent studies. In fact, NGS studies have revealed a higher diversity of taxa and accurate relative abundances in samples coming from different environments, including the vineyard (Peay et al., 2016; Morgan et al., 2017; Jayawardena et al., 2018). Despite these recent advances, culture-independent studies describing the microbial endosphere of grapevines are still scarce.

DNA metabarcoding is a promising tool to investigate the microbial communities present in the wood of grapevines, as it may lead to a new understanding of the complexity that characterizes grapevine trunk diseases (GTD). This cluster of fungal diseases affects primarily the perennial organs of the plants, such as the trunk and roots, however, secondary symptoms may be observed in leaves, bunches and shoots. Overall, GTD cause a loss in vigor, productivity, quality of the yield and lifespan of the plants, with conspicuous economic consequences (Hofstetter et al., 2012; Bertsch et al., 2013; Fontaine et al., 2016; Calzarano and Di Marco, 2018). GTD pathogens are phylogenetically unrelated, belonging to different families, orders and even phyla, although plants infected may reveal similar symptomatology. For example, wood discoloration and necrosis are symptomatology shared by all GTD, whereas the ‘tiger stripes’ pattern in the leaves are attributed to grapevine leaf stripe disease (GLSD) and, by some authors, to Botryosphaeriaceous fungi responsible for the syndrome ‘black dead arm’ (Mugnai et al., 1999; Larignon et al., 2009; Bertsch et al., 2013). Moreover, the simultaneous presence of several possible causal agents in infected grapevines complicates the outline of a clear etiological pattern (Edwards and Pascoe, 2004; Bruez et al., 2016; Mondello et al., 2017). This is especially true in the case of esca, a disease complex consisting of five separate syndromes (brown wood streaking of rooted cuttings, Petri disease, GLSD, white rot and esca proper) in which, according to current literature, several pathogenic fungi play a role (Surico, 2009). These fungi may infect vines in the field, where conidia or other propagules reach fresh pruning wounds and start colonizing the xylem, or during the propagation process in nurseries (Gramaje et al., 2018). The pathogens most frequently associated with the first three syndromes are *Phaeomoniella chlamydospora* and *Phaeoacremonium minimum*, two tracheomycotic ascomycetes; while the latter two syndromes are associated with the presence of the wood rotting basidiomycete *Fomitiporia mediterranea* (white rot), especially common in Europe, or with the simultaneous presence of both tracheomycotic and wood rotting pathogens (esca proper) (Surico, 2009). Along with these three players, other wood pathogens including, but not limited to, members of the Diatrypaceae and Botryosphaeriaceae are often found in symptomatic plants (Edwards and Pascoe, 2004; Hofstetter et al., 2012; Bruez et al., 2014; Travadon et al., 2016). Studies that used the NGS approach to learn more about the grapevine endosphere are scarce (Dissanayake et al., 2018; Jayawardena et al., 2018) and none of them investigated the mycobiome of GTD-affected plants.

This work aims to investigate the fungal communities present in the wood of grapevines, in a vineyard with history of esca proper. Three main objectives were set, namely (1) to characterize the mycobiome of the wood of *V. vinifera* cv Cabernet Sauvignon, in a vineyard located in the Lisbon area (Portugal), using Illumina^®^ NGS; (2) to understand the spatial distribution of the communities present in different areas of perennial wood and in canes (annual wood); (3) to understand whether there is a link between the microbial communities of the wood and the expression of leaf symptoms of esca.

## Materials and Methods

### The Vineyard

This study focuses on a vineyard in Portugal, which ranks 11th in the world for wine production, with a total vineyard area of 195 kha (Aurand, 2017). *V. vinifera* cultivar Cabernet Sauvignon is the most cultivated worldwide, with a total vineyard area of 340 kha. It is considered susceptible to trunk diseases (Darrieutort and Pascal, 2007; Eskalen et al., 2007) and has already been cited in studies concerning the microbial ecology of wood, phyllosphere and grapes (González and Tello, 2011; Bruez et al., 2014; Morgan et al., 2017; Singh et al., 2018).

Field sampling took place in the experimental vineyard (Almotivo) of the Instituto Superior de Agronomia, in Lisbon (38°42′32.7′′N, 9°11′11.5′′W). The vineyard has a density of 3,333 plants/ha, the soil is classified as vertisoil, it is managed under conventional agricultural practices and there is no irrigation system. The selected cultivar was Cabernet Sauvignon grafted on 140 RU rootstock (*Vitis berlandieri* × *Vitis rupestris*), 19 years-old at the moment of sampling (planted in 1998), trained as Cordon Royat Bilateral and spur pruned. The field has a history of esca, with leaf-symptomatic grapevines accounting for less than 1% of the total plants in all recorded years (2015, 2016, and 2017). The selection of the plants used in this experiment was restricted to a block of 450 m^2^.

The immediate surroundings of the vineyard, within a 25 m radius from the perimeter, are characterized by the presence of diverse vegetation. Most of the trees are subject to winter pruning and present exposed heartwood from which wood decay have developed. The majority of the species are listed in Supplementary Table S1.

### Sampling and Experimental Setup

**FIGURE 1:**
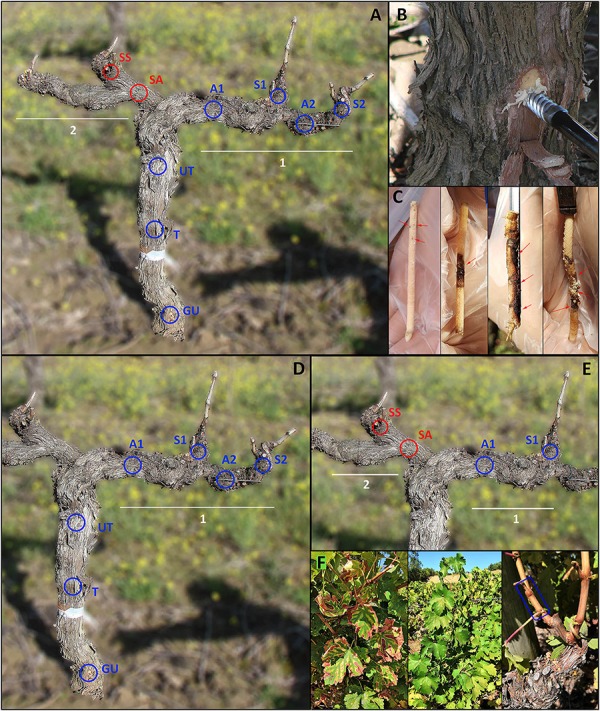
Sampling points in perennial wood or canes of grapevine cv Cabernet sauvignon. (GU) Graft union, (T) Trunk, (UT) Upper trunk, (A1) Arm 1, (S1) Spur 1, (A2) Arm 2, (S2) Spur 2, (SA) Symptomatic arm, (SS) Symptomatic spur. Cordon (1) presented healthy leaves in all ten sampled plants, while cordon (2) presented leaf symptoms, in canes departing from SS, in five of the sampled plants. Red circles indicate wood sampled in proximity of symptomatic leaves, blue circles indicate wood not associated with leaf symptoms. **(A)** Sampling points used to characterize the mycobiome of perennial wood – objective 1 –. **(B)** Sampling procedure involved using a gimlet to drill the wood and extract wood cores. **(C)** Cores of wood extracted with a gimlet (red arrows indicate wood symptomatology). From left to right: brown wood streaking, wood necrosis, extensive wood necrosis, wood decay-white rot-wood necrosis. **(D)** Sampling points used to test the spatial distribution of fungal communities – objective 2 –. **(E)** Sampling points used to examine the mycobiome present in the wood in proximity of symptomatic (AS, SS) and healthy (A1, S1) leaves – objective 3 –. **(F)** From left to right: symptomatic canes sampled from plants with leaf symptoms, asymptomatic canes sampled from plants with no leaf symptoms in either of the cordons or with leaf symptoms in only one of the two cordons; the sampling area for each cane is indicated by the blue rectangle.

Samples of perennial wood (PW) were taken in a non-destructive way, in April 2017, by means of hand-drilling the plants with a gimlet (Figure 1B). The sampling procedure occurred as follows. The bark, on each sampling point, was removed with the use of a knife and the wounds were disinfected with ethanol (70% v/v); the gimlet was placed perpendicularly on the open wounds and manually forced in the wood until it went through the whole width of the plant. This allowed us to extract cores of 19 years-old wood (5 mm of diameter and approximately 60 mm long) which were immediately placed in sterile 15 mL falcon tubes and temporarily stored in ice (Figure 1C). Samples were then transferred to a freezer, freeze-dried and stored at −80°C. After extracting each core of wood, the gimlet was sterilized by dipping it in a sodium hypochlorite solution (0.35 w/w of active chlorine) for 1 min, followed by a rinse with ethanol (70% v/v) and then double-rinsed with sterile distilled water (SDW), in order to minimize cross-contamination.

Canes grown in the 2017 growing season were sampled in September, as annual wood, detaching them with pruning scissors, approximately 3 cm above the spur from which they departed (length of wood sampled: 50 mm; age of wood: 5 months-old; Figure 1F). Each sample was deprived of its bark, frozen, freeze-dried and stored at −80°C until processing.

Concerning the PW, five grapevines with healthy leaves were sampled in 7 precise areas each (*n* = 35, Figure 1D) and five grapevines with symptomatic leaves were sampled in 9 precise areas each (*n* = 45, Figure 1A). The former five plants did not show leaf symptoms of esca during the previous two growing seasons (years 2015 and 2016), while the latter five manifested leaf ‘tiger stripes’ symptoms, only in one of the two cordons (cordon 2; Figure 1A), during the previous growing season (year 2016). The terms ‘asymptomatic’ and ‘symptomatic,’ which will often be encountered in the rest of the text, refer exclusively to leaf symptomatology and not to wood symptomatology (unless specifically stated).

The tissue types corresponding to the nine sampling areas are: ‘Graft Union’ (GU), located approximately (3 ± 1 cm) above the soil, on the graft union; ‘Trunk’ (T), (22 ± 1 cm) above GU; ‘Upper Trunk’ (UT), (22 ± 1 cm) above T; ‘Arm 1’ (A1), was located on the cordon, (36 ± 2 cm) away from UT; the sample point ‘Spur 1’ (S1) is located on the cordon, right below the spur, (10 ± 1 cm) from A1; ‘Arm 2’ (A2), located (22 ± 1 cm) to the right of A1; ‘Spur 2’ (S2), (10 ± 1 cm) from A2 (Figure 1). All canes departing from cordon 1 (S1, S2) did not exhibit leaf symptoms, for all 10 sampled plants. Sampling points (SA, symptomatic arm) and (SS, symptomatic spur) are the equivalent of points (A1) and (S1), but located in cordon 2. In this case, 5 out of the 10 sampled grapevines presented symptoms in the leaves of canes departing from (SS), while the other 5 plants had non-symptomatic leaves.

Only one tissue type was examined in annual wood, namely the ‘Canes,’ where 15 canes were sampled from 10 plants. Five of them came from asymptomatic plants, while the other 10 came from symptomatic plants. Within these 10, 5 canes were leaf-symptomatic (cordon 2, Figure 1), while the other 5 were asymptomatic and sampled in the cordon 1 (Figure 1F).

To address the three objectives of this study, the sample points corresponding to different tissue types were combined as follows. (1) To characterize the mycobiome of the wood of the vineyard, all sample points were taken in consideration (*n* = 80 from PW, *n* = 15 from canes; Figure 1A). (2) To learn about the spatial distribution of the mycobiome in the different areas of the plants, we used tissue types (GU – S2) (*n* = 10 per tissue type, total *n* = 70; Figure 1D), along with asymptomatic canes (*n* = 10). (3) To understand the link between leaf symptoms expression and mycobiome of PW or canes, we created three groups per category. In the category ‘PW,’ group (i) consisted in asymptomatic plants (cordon 1, points ‘A1 and S1’; *n* = 10), group (ii) consisted in symptomatic plants, sampled in the asymptomatic cordon (cordon 1, points ‘A1 and S1’; *n* = 10), group (iii) consisted in symptomatic plants, sampled in the symptomatic cordon (cordon 2, points ‘SA and SS’; *n* = 10; Figure 1E). The same applies to the category ‘canes,’ where group (i) consisted in asymptomatic canes, sampled from asymptomatic plants, group (ii) consisted in asymptomatic canes, sampled from symptomatic plants, group (iii) consisted in symptomatic canes (*n* = 5 per group).

### DNA Extraction, Amplification, Library Preparation and Sequencing

Wood samples were ground to dust using sterile mortars and pestles, aiding the process with liquid nitrogen. An aliquot of ground wood (0.25 ± 0.01 g) of each sample was added to DNA extraction columns (FastDNA^*TM*^ SPIN Kit for Soil, MP Biomedicals^®^ LLC) and total DNA was extracted as described by the kit manufacturer. Three negative controls of the DNA extraction procedure were added.

Among the several possible informative genes (e.g., β-tubulin, elongation factor), the amplicon chosen in this study targeted the Internal Transcribed Spacer ITS1 region, and the primer set selected was ITS1F2 – ITS2 (Gaylarde et al., 2017) with overhang recommended by Illumina. For building libraries, we used a double-step PCR approach as reported by Feld et al. (2015). The full sequence of the primers, including Illumina overhangs, is the following: ITS1F2 (5′-TCGTCGGCAGCGTCAGATGTGTATAAGAGACAG-GAACC WGCGGARGGATCA-3′) and ITS2 (5′-GTCTCGTGGGC TCGGAGATGTGTATAAGAGACAG-GCTGCGTTCTTCATC GATGC-3′) (Gaylarde et al., 2017).

Each first-step PCR reaction contained 12.5 μL of Supreme NZYTaq II 2x Green Master Mix^*TM*^ (NZYtech^*TM*^), 0.5 μL of forward and reverse primers from a 10 μM stock, 1.5 μL of sterile water, and 5 μL of template. Each reaction was pre-incubated at 95°C for 2 min, followed by 40 cycles of 95°C for 15 s, 75°C for 10 s, 55°C for 15 s, 72°C for 40 s; a further extension was performed at 72°C for 10 min.

Second PCR-step for barcoding, fragment-purification by using MagBio beads and Qubit quantification was performed as reported in Gobbi et al. (2019). Final pooling was performed at 10 ng/sample. DNA Sequencing was performed using an in-house Illumina^®^ MiSeq instrument and 2 × 250 paired-end reads with V2 Chemistry.

### Bioinformatics

After sequencing, demultiplex was performed using our Illumina MiSeq platform and the raw data were analyzed using QIIME 2 v. 2018.2 (Caporaso et al., 2010) using the same pipeline described in Gobbi et al. (2019); denoised reads were trimmed 15 bp on the left to remove the adapters and then they were analyzed using DADA2 with the exact sequence variants (EVS) methods (Callahan et al., 2017). Each ESV appears at least twice in the dataset. Singletons were discarded. Taxonomic assignments were performed at 99% identity using qiime feature-classifier classify-sklearn with a Naïve-Bayes classifier trained with UNITE (Nilsson et al., 2013) v7.2 for ITS. To test the three hypotheses underlying this work we separate the frequency table in three sub-tables, which were tested under different conditions. The raw data of this study is available in the European Nucleotide Archive (ENA accession number PRJEB31028).

### Statistics

The frequency table and its taxonomy were combined, converted to biom format in QIIME (Caporaso et al., 2010), then merged with a table of metadata into an S4 object and analyzed in R (v. 3.4.3) using the following packages: phyloseq, v. 1.22.3 (McMurdie and Holmes, 2013); biomformat, v. 1.10.0 (McMurdie and Paulson, 2018), vegan, v. 2.5.2 (Oksanen et al., 2007); ggplot2, v. 3.0.0 (Wickham, 2016); igraph, v. 1.2.2 (Csardi and Nepusz, 2006), MetacodeR, v. 0.2.1.9005 (Foster et al., 2017); adespatial, v. 0.1.1 (Dray et al., 2018); data.table, v 1.10.4.3 (Dowle and Srinivasan, 2017); and microbiome, v. 1.4.0. R code is publicly available at https://github.com/Marieag/EMG/blob/master/vine_microbiome_init.R.

To assess the alpha diversity, Shannon diversity index and Pielou’s evenness were calculated and tested using a one-way ANOVA with *post hoc* Tukey’s HSD (Honestly Significant Difference), determining differences in these indexes between tissue types or tissue groups.

We analyzed the β-dispersion to measure between-sample variances in abundance, computing average distances of the individual samples. The resulting ordination was plotted using the non-metric multidimensional scaling (NMDS) combined with a Jaccard index matrix. These ordinations were also performed with a Bray–Curtis dissimilarity matrix. To assess overall inter-group variance, we performed a PERMANOVA, using a Jaccard distance matrix with 999 permutations. The *post hoc* test for the PERMANOVA (performed on the EVS counts) was done using the adonis function from the vegan package in R.

In order to illustrate this effect size compared to the relative abundance of the taxa, we created differential heat trees using MetacodeR, illustrating the log2 fold change in species abundance. A Wilcoxon Rank Sum test was applied to test differences between the same species in different tissue types or tissue groups, and the resulting *p*-values were corrected for multiple comparisons using FDR, as implemented in MetacodeR. *P*-value threshold was set to 0.05.

## Results

### Sequencing Dataset Description

This dataset, obtained by sequencing, consists of a total of 95 samples. It includes 80 samples collected from different parts of the PW and 15 from canes. All of them represent in total 20 plants, and each one of them counts as an independent biological replicate. This dataset contains 2,805 exact sequence variants which appear a total of 8.184.885 times among all the different samples. The raw tables of EVSs (read counts), for each of the three objectives of this study, are available in Supplementary Data Sheet 1.

### Visual Examination of the Sampled Wood

Before processing the samples for the molecular analysis, wood cores of PW and samples of canes were visually examined to assess the presence of wood symptomatology. Approximately 10% of the wood cores were fully asymptomatic, 75% presented symptoms of tracheomycosis (e.g., brown wood streaking and/or wood necrosis), and 15% showed the presence of white rot, which was always associated with other tracheomycosis symptoms. The examination of the canes wood revealed that 100% of the samples were fully asymptomatic.

### The Wood Mycobiome

The identification of sequences in our dataset revealed an unprecedented diversity. Taxa that were assigned to genus or species level are 289, 50 of them are found in relative abundance (RA) greater than 0.1%, while the remaining 239 are considered rare taxa (RA < 0.1%). Within these 239 taxa, 146 are found in a RA included between 0.1 and 0.01%, while the remaining 93 have a RA lower than 0.01%. The full list of taxa is available in Supplementary Table S2.

The qualitative overview of the wood mycobiome will focus mainly on the 30 most abundant taxa in PW and the 12 most abundant in canes, which account for 79.1 and 80.8% of the total RAs, respectively (Table 1), while the remaining percentages represent unidentified taxa or fungi found in lower abundances. Within this group of taxa, five genera and nine species of fungi are described for the first time as part of the grapevine wood mycobiome, while the other 18 taxa have already been reported in literature (Table 1).

**TABLE 1:** List of most abundant taxa, identified to genus or species level, found in grapevine wood.

**Phylum**	**Family**	**Species**	**Relative abundance (%)**		Ecology in wood ^*^	**Presence in different tissue type**			
			**PW**	**C**		**GU/T/UT**	**A1/A2**	**S1/S2**	**C**
Ascomycetes (66.7–76.3)	Biatriosporaceae (0.6– 0)	*Biatriospora mackinnonii*^‡^	0.6	–	E^a^	−/−/−	−/−	−/−	−
	Bionectriaceae (0.4–0)	*Clonostachys rosea*	0.4	–	E, S, P^*b*^	−/−/−	−/−	−/+	−
	Davidiellaceae (2.0–8.2)	*Cladosporiu m* sp.	1.9	8.2	E, S^*b*^	+/+/+	+/+	+/−	+
	Diaporthaceae (<0.1–0.8)	*Diaporthe* sp.	<0.1	0.8	E, S, P^*b*^	−/−/−	−/−	−/−	+
	Diatrypaceae (2.6–0)	*Anthostoma gastrinum*^†^	0.9	–	S, P^c,d^	+/+/+	+/+	+/−	−
		*Eutypa lata*	0.7	–	P^*b*^	−/−/−	−/−	−/+	−
		*Eutypa leptoplaca*	0.9	–	P^*b*^	−/−/+	−/−	+/+	−
	Dothioraceae (0.4–4.0)	*Aureobasidium pullulans*	0.4	4.0	E, S^*b*^	−/+/+	+/+	−/−	+
	Glomerellaceae (<0.1–0.7)	*Colletotrichum* sp.	<0.1	0.4	P^*b*^	−/−/−	−/−	−/−	−
	Herpotrichiellaceae (27.1–3.9)	*Exophiala xenobiotica*^†^	0.5	–	na	−/−/−	−/+	+/+	−
		*Phaeomoniella chlamydospora*	25.8	3.9	P^d^	+/+/+	+/+	+/+	+
	Hypocreales (0.3–0.2)	*Acremonium* sp.	0.2	–	E^e^	−/−/−	−/−	−/+	−
	Lophiostomataceae (3.8–0)	*Angustimassarina acerina*^†^	0.5	–	S^f^	−/−/+	+/+	+/+	−
		*Lophiostoma* sp.	2.7	–	E, S^*b*^	+/+/−	+/−	+/−	−
		*Lophiostoma cynaroidis*^†^	0.3	–	E^g^	−/+/−	−/−	−/−	−
		*Lophiotrema rubi*	0.3	–	na	+/−/−	−/−	−/−	−
	Massarinaceae (0.5–0)	*Massarina* sp.	0.5	–	E^h^	+/+/+	−/−	+/−	−
	Mycosphaerellaceae (3.3–9.4)	*Ramularia* sp.	3.1	9.4	na	+/+/+	+/+	+/+	+
	Pleomassariaceae (2.9–0)	*Trematosphaeria pertusa*^‡^	2.9	–	S^i^	+/+/−	−/+	+/+	−
	Pleosporaceae (3.9–14.8)	*Alternaria* sp.	3.2	14.6	E^*b,j*^	+/+/+	+/+	+/+	+
			PW	C					
	Saccharomycetaceae (10.9–31.8)	*Debaryomyces prosopidis*^‡^	10.4	31.5	na	+/+/+	+/+	+/+	+
	Xylariaceae (0.7–0)	*Lopadostoma meridionale*^‡^	0.3	–	S^k^	−/−/−	−/−	−/−	−
		*Lopadostoma quercicola*^‡^	0.4	–	S^k^	+/−/−	−/−	−/−	−
Basidiomycetes (26.7–18.8)	Filobasidiaceae (0.2–0.2)	*Filobasidium magnum*^†^	0.1	0.2	na	+/−/−	−/+	−/−	−
	Hymenochaetaceae (15.2–0)	*Fomitiporia* sp.	14.6	–	P^d^	+/+/+	+/+	+/+	−
		*Fomitiporia mediterranea*	0.2	–	P^d^	−/+/−	+/−	−/−	−
		*Inonotus hispidus*	0.3	–	S, P^l^	+/−/−	−/+	−/−	−
	Malasseziaceae (0.3–0.3)	*Malassezia restricta*^‡^	0.2	0.2	na	+/−/−	−/−	−/−	+
	Psathyrellaceae (0.5–0)	*Psathyrella* sp.	0.5	–	S^m^	+/−/−	−/−	−/−	−
	Sporidiobolaceae (0.6–0)	*Rhodotorula mucilaginosa*^†^	0.4	<0.1	S^n^	−/+/+	+/+	−/−	−
	Tremellaceae (6.4–8.3)	*Cryptococcus* sp.	2.7	7.6	E, S^*b*^	+/+/+	+/+	+/+	+
		*Cryptococcus heimaeyensis*^†^	0.3	–	–	−/−/+	−/−	−/−	−
		*Cryptococcus victoriae*^†^	2.9	0.7	–	+/+/+	+/+	+/+	+
		Total	**79.1**	**80.8**					

The list includes the 30 most abundant taxa found in perennial wood and the 12 most abundant in canes, for a total of 32 taxa. The numbers between brackets represent the relative abundance of that Phylum or Family in perennial wood or canes (PW% – C%) based on the table created to address objective (1). The ecology of the identified taxa in wood of grapevines or of other plants is shown based on available literature (E, endophyte; S, saprophyte; P, pathogen; na, unknown ecology). The presence of taxa in different tissue types is based on the table created to address objective (2), (+) indicates presence (RA ≥ 0.1%), (−) indicates absence or presence in RA < 0.1%. (GU) Graft union, (T) Trunk, (UT) Upper trunk, (A1) Arm 1, (S1) Spur 1, (A2) Arm 2, (S2) Spur 2 and (C) Canes. ^*^References. ^*a*^(Kolařík et al., 2017), ^*b*^(Jayawardena et al., 2018), ^*c*^(Haynes, 2016), ^*d*^(Gramaje et al., 2018), ^*e*^(González and Tello, 2011), ^*f*^(Thambugala et al., 2015), ^*g*^(Xing et al., 2011), ^*h*^(Casieri et al., 2009), ^*i*^(Suetrong et al., 2011), ^*j*^(Pancher et al., 2012), ^*k*^(Jaklitsch et al., 2014), ^*l*^(González et al., 2009), ^*i*^(Bruez et al., 2016), ^*n*^(Haňáčková et al., 2017). ^‡^First report of genus and species in grapevine wood. ^†^First report of species in grapevine wood.

The community encountered in PW is characterized by the presence of both ascomycetes and basidiomycetes (66.7% and 26.7% RA), with high abundances of tracheomycotic pathogen *P. chlamydospora* (25.8%) and white rot agent *Fomitiporia* sp. (14.6%), two organisms directly associated with esca proper and other esca-related syndromes. Among all sampled wood cores of PW (*n* = 80), *P. chlamydospora* was present in 68 of them (85%; RA > 0.1%), while *Fomitiporia* sp. in 58 (64%; RA > 0.1%) or 14 (17.5%; RA > 35%). Other GTD pathogens among the 30 most abundant taxa are *Eutypa lata* (0.7%) and *Eutypa leptoplaca* (0.9%), within the Diatrypaceae. Other members of this family are *Anthostoma gastrinum* (0.9%), a potential wood pathogen, as well as *E. flavovirens*, *Eutypella citricola*, and *Cryptovalsa ampelina*, identified as rare taxa. Members of the Botryosphaeriaceae (e.g., *Diplodia pseudoseriata*, *Neofusicoccum parvum*, *Neofusicoccum australe*), *Ilyonectria* sp. and *Neonectria* sp. are also found, although represented only as rare taxa (Supplementary Table S2). Decay agents, such as *Fomitiporia* sp., *F. mediterranea* (0.2%) and *I. hispidus* (0.3%), were also identified in this study, along with several others represented in minor abundances (e.g., *Fomitiporella* sp.). Among the endophytes and saprophytes, *Alternaria* sp. (3.2%), *Cladosporium* sp. (1.9%), *Aureobasidium pullulans* (0.4%), and *Psathyrella* sp. (0.5%) are the most abundant. Several other genera or species, identified for the first time in association with grapevine wood, amount to 14 taxa out of the 33 most abundant in PW or canes (Table 1).

Canes were also colonized by both ascomycetes (76.3%) and basidiomycetes (18.8%). The most abundant taxa are endophytic and saprophytic fungi, with *Alternaria* sp. (14.6%), *Ramularia* sp. (9.4%) and *Cladosporium* sp. (8.2%) being among most abundant, as well as other species reported for the first time (e.g., *Debaryomyces prosopidis*; Table 1). Only two wood pathogens are present in moderate abundances in canes, namely *P. chlamydospora* (3.9%) and *Diaporthe* sp. (0.8%), while other pathogenic agents are found in minor abundances (RA < 0.2%; e.g., *N. australe*).

The core mycobiome, namely the taxa shared between PW and canes, is constituted by 44 taxa. Only 10 taxa are unique to canes and the remaining 235 are unique to PW. All the 10 unique taxa found in canes are considered rare taxa, as their RAs are lower than 0.1% of the total, while among the many taxa unique to PW we find organisms belonging to the Hymenochaetaceae, Lophiostomataceae, Pleomassariaceae, Xylariaceae and numerous others (Supplementary Table S2).

### Diversity and Spatial Distribution of the Mycobiome

#### Alpha Diversity

**FIGURE 2:**
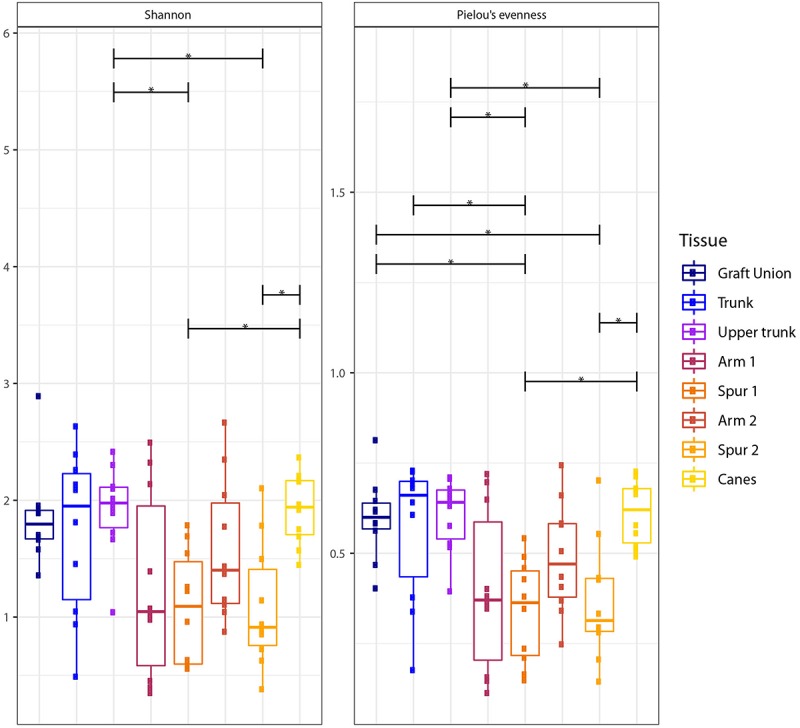
Box plots of diversity indexes (Shannon, Pielou’s evenness) of the fungal communities present in different sampling areas of perennial wood (Graft Union, Trunk, Upper trunk, Arm 1, Spur 1, Arm 2, and Spur 2) and canes. Vertical boxes denote the median, the upper and lower quartiles, and the extremes of data. The black, horizontal brackets at the top of the figure denote statistical comparisons of the two tissues at each end of the bracket, calculated using a one-way ANOVA with *post hoc* Tukey’s HSD. Statistical differences are shown by asterisks, where *P* < 0.05 = ^*^.

The Shannon (*H*′) and Pielou’s (*J*′) indexes vary significantly among tissue types, according to Tukey’s HSD (Figure 2 and Table 2). The Shannon diversity analysis reveals that one sample point, namely the Upper Trunk, differs from both the spur points S1 and S2 (*P* < 0.05). The spur tissue is also significantly different from canes (*P* < 0.05). No differences are observed when comparing GU, T, UT, the two sample points in the arm (A1 and A2) and canes. Exact *p*-values of the significant differences are available in Supplementary Table S3.

**TABLE 2:** Shannon diversity (*H*′) and Pielou’s evenness (*J*′) indexes of the spatial distribution analysis (objective 2) and the mycobiome analysis in the wood associated with symptomatic leaves (objective 3).

	**Shannon (*H***′**)**		**Pielou’s evenness (*J***′**)**	
	**Value**	***P* < 0.05**	**Value**	***P* < 0.05**
**Spatial distribution (objective 2)**				
Graft Union (GU)	1.85	–	0.60	S1, S2
Trunk (T)	1.73	–	0.57	S1
Upper trunk (UT)	1.92	S1, S2	0.61	S1, S2
Arm 1 (A1)	1.26	–	0.40	–
Spur 1 (S1)	1.08	UT, C	0.34	GU, T, UT, C
Arm 2 (A2)	1.58	–	0.49	–
Spur 2 (S2)	1.09	UT, C	0.37	GU, UT, C
Canes (C)	1.93	S1, S2	0.61	S1, S2
**Leaf symptoms (objective 3)**				
**Perennial wood**				
Asymptomatic arm in asymptomatic plant	1.29	–	0.41	–
Asymptomatic arm in symptomatic plant	1.05	–	0.33	–
Symptomatic arm	1.25	–	0.39	–
**Canes**				
Asymptomatic cane in asymptomatic plant	2.04	–	0.63	–
Asymptomatic cane in symptomatic plant	1.81	–	0.59	–
Symptomatic cane	2.09	–	0.68	–

**Objective 2 aims to asses differences between tissue types sampled in different areas of grapevines; objective 3 aims to asses differences between non-leaf-symptomatic and leaf-symptomatic woody tissue, in perennial wood or canes. Tissue types significantly different, according to Tukey’s HSD (P < 0.05), are listed under the column ‘P < 0.05.’**

Different tissue types also vary in mycobiome evenness, with fungal communities of canes, GU, T and UT being more evenly distributed than those of the spurs (*P* < 0.05; Figure 2). All other tissue types examined do not differ in evenness (Figure 2).

#### Beta Diversity

**FIGURE 3:**
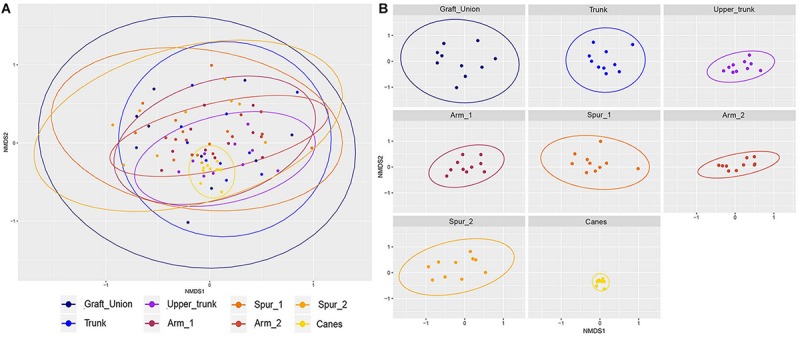
Non-metric multidimensional scaling (NMDS) plots based on Jaccard’s index. Fungal communities present in different tissue types in grapevine. **(A)** Shows all samples ordinated together, while **(B)** is the same data, split up per tissue type. Ellipses illustrate the multivariate normal distribution of samples within the same tissue type.

The Jaccard’s index, when visualized in a non-metric multidimensional scaling (NMDS) plot, shows a considerable overlap for different tissue types (Figure 3A). The PERMANOVA indicates significant difference between groups (*P* < 0.001), but looking at the ordination, the difference is arguably in the clustering of the observations, rather than a distinct difference in sample composition. A pattern emerges when examining each tissue type separately (Figure 3B). We observe a reduction in between-sample variability starting from the GU to T and until UT. The UT variability is very similar to that of both the Arm points (A1 and A2), while the Spurs (S1 and S2) have higher between-sample variability, when compared to the Arms, but similar to one another. Concerning the canes, the variability of this tissue type is very low (Figure 3B). Bray–Curtis test revealed a similar profile and significant differences (*P* < 0.001; data not shown). Jaccard and Bray–Curtis matrixes are available in the Supplementary Materials.

#### Mycobiome Composition and Differentially Represented Taxa

**FIGURE 4:**
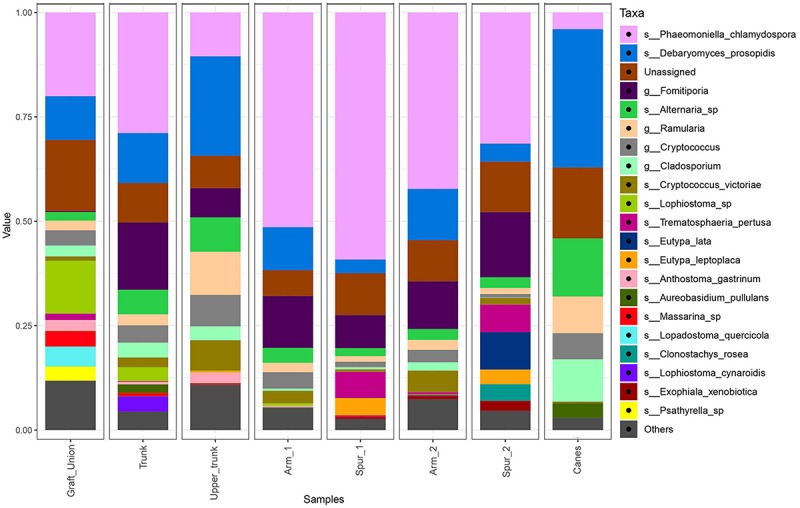
Barplots of the relative abundance of the 20 most abundant taxa identified to species (s_) or genus (g_) level, found in different sampling areas of the stem and in the canes of grapevine. ‘Unassigned’ are taxa identified to a lower taxonomic level than genus, ‘Others’ are taxa not included in the 20 most abundant.

**FIGURE 5:**
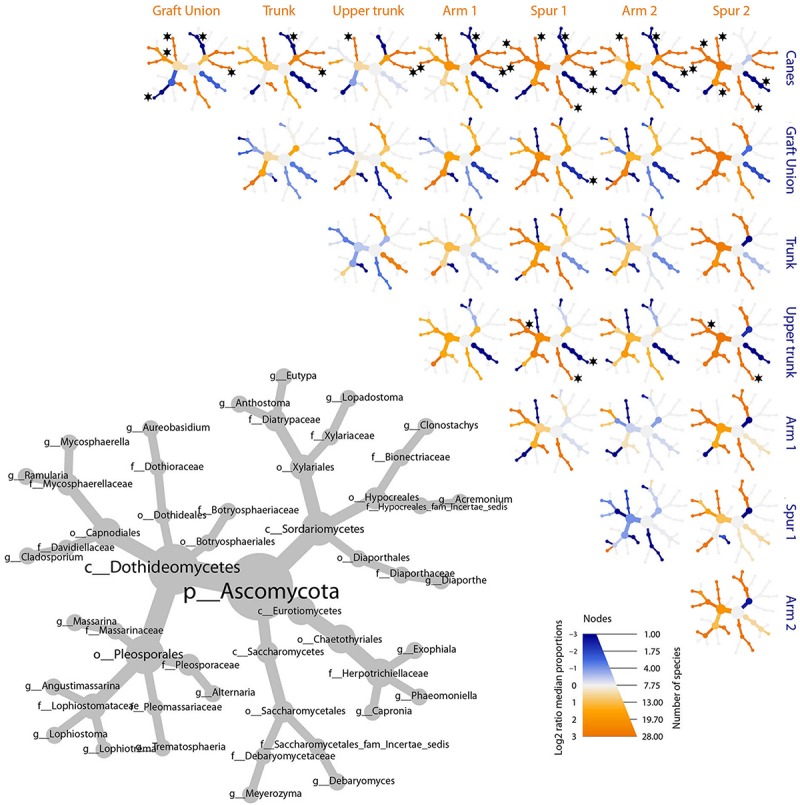
Differential heat tree matrix depicting the change in taxa abundance between different tissue types, for ascomycetes, represented in the dataset (RA > 0.01%). The size of the individual nodes in the gray cladogram depicts the number of taxa identified at that taxonomic level. The smaller cladograms show pairwise comparisons between each tissue type: an orange node indicates a higher abundance of the taxon in the tissue type stated on the abscissa, than in the tissue type stated on the ordinate. A blue node indicates the opposite. Taxa identified as statistically differently represented, according to the Wilcoxon test, are tagged with a black star.

**FIGURE 6:**
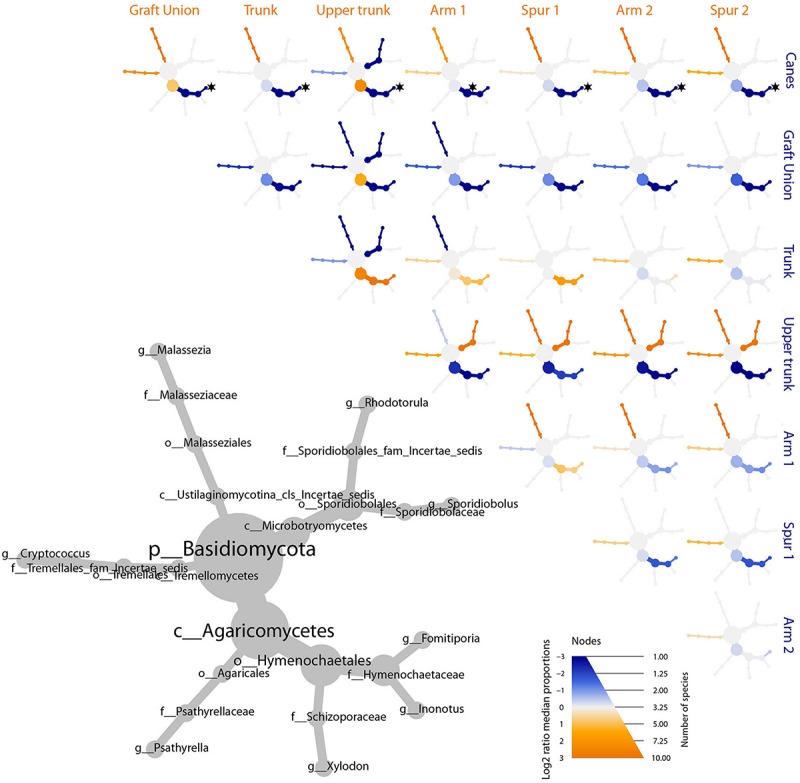
Differential heat tree matrix depicting the change in taxa abundance between different tissue types, for basidiomycetes, represented in the dataset (RA > 0.01%). The size of the individual nodes in the gray cladogram depicts the number of taxa identified at that taxonomic level. The smaller cladograms show pairwise comparisons between each tissue group: an orange node indicates a higher abundance of the taxon in the tissue group stated on the abscissa, than in the tissue group stated on the ordinate. A blue node indicates the opposite. Taxa identified as statistically differently represented, according to the Wilcoxon test, are tagged with a black star.

The bar plot in Figure 4 shows the relative abundance of the 20 most abundant taxa in different tissue types, giving an overview of the presence/absence of taxa and their differential representation. For example, *E. lata* and *Acremonium* sp. are present exclusively in the wood below the spurs, while *Trematosphaeria pertusa* was detected in the same tissue type and in the graft union. *Fomitiporia* sp., abundant in most of PW, is nearly absent in the graft union. This tissue type contains three unique taxa, *Psathyrella* sp., *Lophiotrema rubi* and *Lopadostoma quercicola*, as well as other taxa which are present in higher abundances when compared to the rest of the plant, such as *Lophiostoma* sp. and *Massarina* sp. The heat trees shown in Figures 5, 6 give a thorough view of the differently abundant taxa, when comparing each tissue type, for taxa with RAs > 0.1% (*n* = 50). The majority of the statistical differences observed, for both ascomycetes and basidiomycetes, concern the comparison between canes and all other tissue types of PW. Among the ascomycetes, canes present lower abundance of *P. chlamydospora* and Diatrypaceae, and higher abundance of *A. pullulans*, *D. prosopidis*, *Diaporthe* sp., Capnodiales, depending on the woody tissues compared.

### Mycobiome and Leaf Symptoms

#### Alpha and Beta Diversity

**FIGURE 7:**
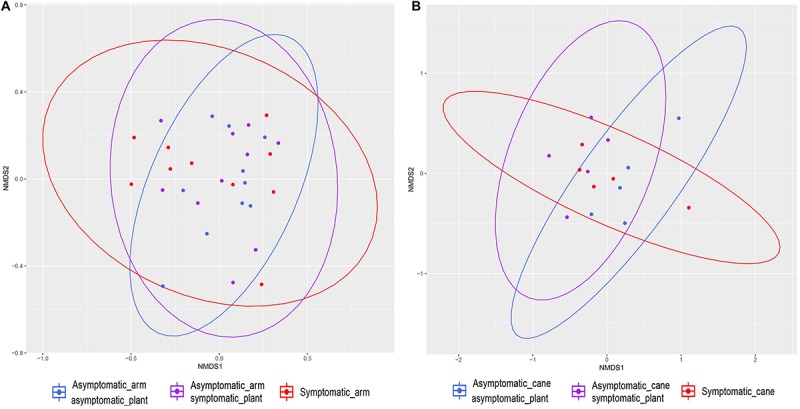
Non-metric multidimensional scaling (NMDS) plots based on Jaccard’s index. Fungal communities present in different tissue types in grapevine. **(A)** Communities found in the perennial wood in proximity of symptomatic leaves (‘Symptomatic_arm’) or of asymptomatic leaves, either in symptomatic plants (‘Asymptomatic_arm symptomatic_plant’) or in asymptomatic plants (‘Asymptomatic_arm asymptomatic_plant’). **(B)** Communities found in canes with manifested leaf symptoms (‘Symptomatic_cane’) or asymptomatic, but coming from symptomatic plants (‘Asymptomatic_cane symptomatic_plant’) or asymptomatic plants (‘Asymptomatic_cane asymptomatic_plant’). Ellipses illustrate the multivariate normal distribution of samples within the same tissue group.

The examination of the mycobiome of PW in proximity of leaves that exhibited ‘tiger stripes’ symptoms revealed no significant differences in term of Shannon diversity (*H*′) and evenness (*J*′), when compared with the fungal communities in the wood in proximity of non-symptomatic leaves, both in symptomatic and asymptomatic vines, according to Tukey’s HSD (*P* > 0.05; Table 2 and Supplementary Figure S1). The same results were obtained when comparing the mycobiome of canes presenting leaf symptoms or non-symptomatic leaves, in both symptomatic and asymptomatic plants. The Jaccard indexes, when plotted in a NMDS matrix, reveal overlapping communities with a very similar between-sample variability, suggesting an overall similarity in beta diversity (Figure 7). In fact, the PERMANOVA analysis revealed no statistical differences among the three tissue groups in both PW (*P* = 0.067) and canes (*P* = 0.429).

#### Mycobiome Composition and Differentially Represented Taxa

**FIGURE 8:**
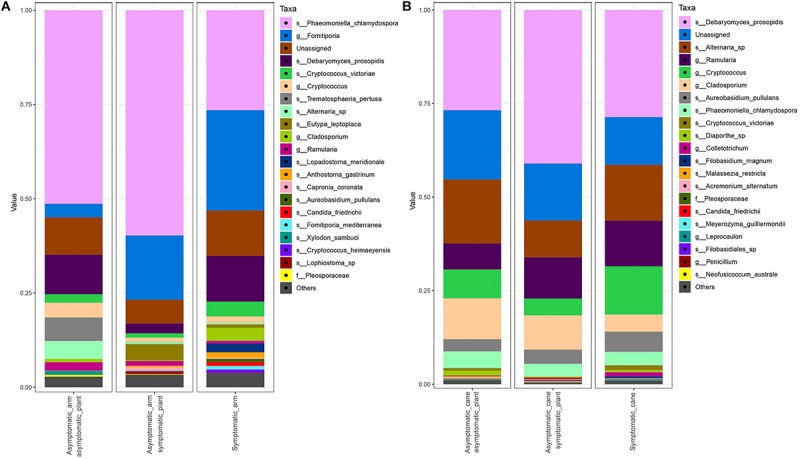
Barplots of the relative abundance of the 20 most abundant taxa identified to species (s_), genus (g_) or family (f_) level. ‘Unassigned’ are taxa identified to a lower taxonomic level than family. ‘Others’ are taxa not included in the 20 most abundant. **(A)** Communities found in the PW in proximity of symptomatic leaves (‘Symptomatic_arm’) or of asymptomatic leaves, either in symptomatic plants (‘Asymptomatic_arm symptomatic_plant’) or in asymptomatic plants (‘Asymptomatic_arm asymptomatic_plant’). **(B)** Communities found in canes with manifested leaf symptoms (‘Symptomatic_cane’) or asymptomatic, but coming from symptomatic (‘Asymptomatic_cane symptomatic_plant’) or asymptomatic plants (‘Asymptomatic_cane asymptomatic_plant’).

The mycobiome of PW in proximity of symptomatic or non-symptomatic leaves is characterized by high abundances of *P. chlamydospora*, *Fomitiporia* sp., and *D. prosopidis* (Figure 8A). The most frequent taxa of known GTD-associated pathogens are presented in Table 3.

**TABLE 3:** Relative abundances (RA) of genera or species of fungi known to be involved in GTD, encountered in the perennial wood (PW) or canes, relative to objective (3).

**Genus/Species**	**Pathogens RA in PW**			**Pathogens RA in canes**		
	
	**SYM**	**ASYM-SYM**	**ASYM**	**SYM**	**ASYM-SYM**	**ASYM**
**Ascomycetes**						
Anthostoma gastrinum^*^	1.3	0.7	–	–	–	–
*Diaporthe* sp.	–	–	<0.1	0.6	<0.1	1.2
*Eutypa lata*	0.2	0.1	<0.1	–	–	–
*Eutypa leptoplaca*	0.7	3.7	<0.1	–	–	–
*Neofusicoccum parvum*	0.1	–	–	–	–	–
*Neofusicoccum australe*				–	0.2	–
*Phaeomoniella chlamydospora*	21.8	50.1	43.1	3.5	3.4	4.9
**Basidiomycetes**						
*Fomitiporia* sp.	34.9	24.1	7.7	–	–	–
*Fomitiporia mediterranea*	0.9	<0.1	0.1	–	–	–
**Total Ascomycetes**	22.7	53.9	43.1	4.1	3.6	6.1
**Total Basidiomycetes**	35.8	24.1	7.8	–	–	–
**TOTAL**	**58.5**	**78.0**	**50.9**	**4.1**	**3.6**	**6.1**

Three groups of wood were examined, namely the PW in proximity of symptomatic leaves (SYM); asymptomatic leaves, but in vines that presented leaf symptoms (ASYM-SYM); asymptomatic leaves, in vines that did not present leaf symptoms (ASYM). The same groups were examined in canes, namely canes with manifested leaf symptoms (SYM); asymptomatic canes, but in vines that presented symptomatic leaves (ASYM-SYM); asymptomatic canes, in vines that did not present symptomatic leaves (ASYM). Taxa listed are found in RA > 0.01% of the total dataset, other known wood pathogens (RA < 0.01%) are not included. ^*^Genus Anthostoma has been associated to pathogenicity in grapevine wood, but not A. gastrinum, being this the first report of this species in grapevine wood. The RAs of this taxon were not calculated in the Total(s).

**FIGURE 9:**
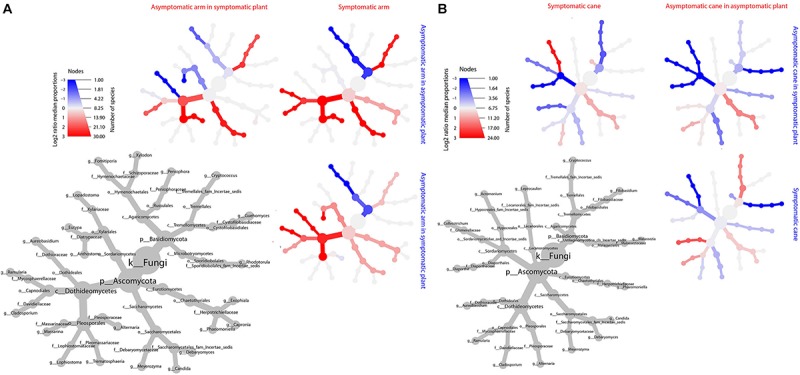
Differential heat tree matrix depicting the change in taxa abundance between different tissue groups, perennial wood **(A)** and canes **(B)**, represented in the dataset (RA > 0.01%). The size of the individual nodes in the gray cladogram depicts the number of taxa identified at that taxonomic level. The smaller cladograms show pairwise comparisons between each tissue group, with the color illustrating the log2 fold change: a red node indicates a higher abundance of the taxon in the tissue group stated on the abscissa, than in the tissue group stated on the ordinate. A blue node indicates the opposite. No taxa were identified as statistically differently represented, according to the Wilcoxon test.

The MetacodeR analysis revealed no statistical differences among taxa for their differential abundance in the three tissue groups compared in PW and in canes. Nevertheless, trends of change are present for some taxa, and they are shown in the heat trees in Figure 9.

The PW in proximity of non-symptomatic leaves in non-symptomatic plants presents higher abundances of *Ramularia* sp., *Cladosporium* spp., *Alternaria* sp., *Debaryomyces* sp., *Cryptococcus* sp. and, to a lower extent, *P. chlamydospora*; while *Fomitiporia* spp. is under-represented, when compared with the wood near symptomatic leaves. When comparing the PW in proximity of non-symptomatic and symptomatic leaves, both collected from a symptomatic vine, similar trends are observed. In fact, *Ramularia* sp., members of the Pleosporales and *Aureobasidium pullulans* are over-represented in the former, along with a minor over-representation of the other taxa previously mentioned. Also, in this case a trend of underrepresentation is observed for *Fomitiporia* spp. Some differences are also observed when comparing the PW near asymptomatic leaves coming from either asymptomatic or symptomatic plants. In the former we observe an over-representation of *Alternaria* sp., *Debaryomyces* sp. and *Cryptococcus* spp., and an under-representation of *A. pullulans* and *Anthostoma* sp. (Figure 9A).

Fungal communities found in canes are characterized by similar relative abundances, among the most abundant taxa, for all three groups analyzed (Figure 8B). Several trends of variation are shown in Figure 9B, involving primarily genera *Malassezia*, *Cryptococcus*, *Acremonium*, and *Diaporthe*.

## Discussion

### Characterization of the Mycobiome in Perennial Wood and Canes

Research performed over the past decades on the grapevine mycobiome, using both culture-dependent and independent approaches, revealed over 900 fungal taxa (Jayawardena et al., 2018). The richness of the wood mycobiome in esca infected vineyards was estimated to be 88 taxa in Montpellier, France (Travadon et al., 2016); 85 species in Bordeaux, France, from 108 single strand conformation polymorphism (SSCP) profiles (Bruez et al., 2014); and 150 operational taxonomic units (OTUs) in Switzerland (Hofstetter et al., 2012). This study, the first using a NGS approach, reveals an even greater richness (289 taxa), adding numerous fungi to the known list of 900+ known taxa. Several factors may play a role in shaping the fungal community composition, such as location, cultivar and age of the plants (Travadon et al., 2016; Dissanayake et al., 2018). This is the primary reason why we expect that using NGS to assess the diversity of the wood mycobiome in other vineyards will considerably increase the number of fungal species found in association with grapevine wood.

Interestingly, 239 out of the 289 taxa (80%) are rare taxa, namely taxa that are detected at relative abundances lower than 0.1%. The ecology of several of them is known (e.g., *Trichoderma* spp., *Neofusicoccum* spp., *Ilyonectria liriodendri*; Jayawardena et al., 2018), while that of many others needs to be assessed (e.g., *Candida* spp., *Cryptococcus* spp., *Ganoderma* spp., and *Pyrenochaeta* spp.), moreover, the extent to which they contribute to the grapevine-mycobiome and fungus–fungus interactions remains to be elucidated. Hypothetically, grapevines perennial wood may function as a reservoir of fungal diversity, where species that are found in minor abundances may thrive under specific environmental conditions (e.g., extreme weather conditions, mechanical injuries), leading to positive or negative effects for the plants wellbeing.

Unsurprisingly, within this massive richness, numerous fungi associated with GTD were identified, both as frequent (e.g., *P. chlamydospora*, *Fomitiporia* sp., and *Eutypa lata*) and rare taxa (*Neofusicoccum* spp., *E. citricola*, *Fomitiporella* sp., *Diplodia* sp.). This agrees with previous reports showing that esca-affected plants may host numerous other wood pathogens (Rumbos and Rumbou, 2001; Edwards and Pascoe, 2004). However, *Phaeoacremonium* spp., a genus of tracheomycotic fungi often involved in esca-associated syndromes (Mostert et al., 2006), was not detected. The vineyard under study, as well as other esca-symptomatic fields examined in previous works (Hofstetter et al., 2012; Bruez et al., 2014; Travadon et al., 2016), are dominated by wood pathogens (e.g., *P. chlamydospora* or Botryosphaeriaceae), and the presence of decay agents was confirmed in all studies. On the other hand, the wood mycobiome of healthy vineyards, characterized by other authors, is dominated by endophytes and occasionally saprobes, some of which hold potential for biological control of wood pathogens, with genera *Trichoderma*, *Aureobasidium*, *Acremonium*, *Cladosporium*, *Alternaria*, and *Epicoccum* being predominant (González and Tello, 2011; Pancher et al., 2012; Dissanayake et al., 2018). Despite the fact that wood pathogens were encountered, in minor abundance, also in healthy vineyards (González and Tello, 2011; Pancher et al., 2012), decay agents were not reported. Unfortunately, previous studies did not report data on rare taxa, therefore it is not possible to establish if other wood pathogens – including decay agents – were present but unable to thrive (e.g., antagonistic interactions with other fungi, strong plant defenses) or if they were completely absent.

The differences observed between PW and canes (e.g., alpha diversity, beta dispersion, taxa composition and abundance), some of which have also been observed in a previous study in grapevines (Hofstetter et al., 2012) and other plants (Qi et al., 2012), can be due to two main factors. First factor is the time that fungi had to colonize PW (up to 19 years in the present study), which is also subjected to yearly pruning, leaving wounds that are the optimal entry point for colonizers. The canes had approximately 5 months of age from bud-burst to sampling and no wounding occurred in the shoots (e.g., summer pruning). A second factor that explains the these differences is the tissue-specificity, as considerable anatomical, biochemical, and physiological differences characterize perennial wood and annual wood, which may prevent some fungi, such as decay agents, from colonizing the latter. In fact, the absence of decay agents in canes (e.g., *Fomitiporia* sp., *I. hispidus*, and *Fomitiporella* sp.) observed in this study, and also reported in canes and nursery propagation material in other studies (Rumbos and Rumbou, 2001; Halleen et al., 2003; Casieri et al., 2009; Hofstetter et al., 2012), suggests that the infection by these pathogens occurs exclusively in older wood and under field conditions.

A brief mention of the five genera identified for the first time in grapevines wood follows.

#### Debaryomyces

*Debaryomyces hansenii* has been isolated from shoots of *Sequoia sempervirens* and from the soil under the tree (Middelhoven, 2003), as well as from white and brown rot of several woody species (González et al., 1989). *Debaryomyces* sp. was also detected in slime fluxes of *Prosopis juliflora*, a deciduous woody plant, as well as in insects associated with it (*Drosophila carbonaria* and *Aulacigaster leucopeza*) (Ganter et al., 1986). Molecular analysis identified some of these strains as a new species, *D. prosopidis* (Phaff et al., 1998), which is the only record of this organism in literature. Concerning the vineyard, *Debaryomyces* sp. has been identified on grape berries (Jara et al., 2016) and during wine fermentation (Varela and Borneman, 2017).

#### Trematosphaeria

*Trematosphaeria pertusa*, from this study, is known to grow on the surface of decaying terrestrial wood (Suetrong et al., 2011), however, it has also been retrieved from wood of *Fagus sylvatica* and *Pinus sylvestris* submerged in a river (Kane et al., 2002). Terrestrial woody hosts of *T. pertusa* include also *Fraxinus excelsior*, *Fagus* sp. and *Platanus* sp.

#### Lopadostoma

Members of the Xylariaceae are typically saprobes, despite some of them are endophytes and others are plant pathogens (Mehrabi and Hemmati, 2015). To date, due to the lack of studies on this genus, the ecology of *Lopadostoma* remains to be fully understood. There are 12 species described in this genus, most of which have been isolated from hosts *Quercus* spp. or *F. sylvatica* (Jaklitsch et al., 2014).

#### Biatriospora

This genus contains species that have been isolated as endophytes of both terrestrial and marine-associated plants (Kolařík et al., 2017), as well as from lichens, as an endolichenic fungus (Zhou et al., 2016). Known hosts of this genus, in temperate climate, are *Ulmus* spp. and *Acer pseudoplatanus*, along with several other hosts from tropical climates (Haňáčková et al., 2017; Kolařík et al., 2017). *Biatriospora* sp. is a source of bioactive compounds (heptaketides) with antifungal properties (Zhou et al., 2016) and preliminary studies suggest some potential in biological control (Haňáčková et al., 2017). *Biatriospora mackinnonii*, from this study, has been isolated from terrestrial plant material and it has also been linked with human mycetoma, a skin disease (Ahmed et al., 2014; Kolařík et al., 2017).

#### Malassezia

*Malassezia restricta*, from this study, has been identified as endophyte of both woody and herbaceous plants (e.g., *Eucalyptus* sp., *Populus deltoides*, *Spiranthes spiralis*, *Solanum tuberosum*) as well as found on orchid roots, and in soil (Connell and Staudigel, 2013; Abdelfattah et al., 2016; Miguel et al., 2017).

It is important to mention the genera *Cryptococcus*, *Angustimassarina*, and *Exophiala*, found in this study, which have been previously associated to grapevines wood just once, either as saprobes or endophytes, in a culture-independent study (Jayawardena et al., 2018). Lastly, species of *Ramularia* have been isolated from leaves (both as epiphytes and endophytes) of several hosts, including *Platanus* sp., *Prunus cerasus*, and *V. vinifera* (Bakhshi and Arzanlou, 2017), although never in association with wood.

Among the genera and species that were found associated with grapevines for the first time in this study, some have previously been detected in the endosphere or phyllosphere of other woody plants that were found in the proximities of the vineyard used in the present study (Supplementary Table S1). For example, *Lopadostoma* spp. and *A. gastrinum* were reported in *Quercus* (Jaklitsch et al., 2014; Haynes, 2016), *Rhodotorula mucilaginosa* and *T. pertusa* in *Fraxinus* (Suetrong et al., 2011; Haňáčková et al., 2017), and *M. restricta* in *Eucalyptus* (Miguel et al., 2017). This suggests that the fungi present in the endosphere and phyllosphere of the flora in proximity of a vineyard might influence the composition of the mycobiome of grapevines wood, acting as a reservoir of multi-host fungi, with wind, rain and insects being possible vectors for mycobiome exchange.

### Spatial Distribution of the Fungal Communities

The concept of spatial distribution and tissue specificity in woody and herbaceous plants is not new in microbial ecology (Wearn et al., 2012; Miguel et al., 2017; Singh et al., 2017; Kovalchuk et al., 2018). However, studies that investigated the spatial distribution of fungal communities that colonize different areas or tissues in the wood of adult grapevines are scarce (Bruez et al., 2014; Travadon et al., 2016), and none of them used a NGS approach. When examining the relative abundances of identified taxa with NGS, it is important to remember that the ITS marker, our target amplicon, can be found in multiple copies in the genome of fungi and the number of copies may vary considerably from species to species (Schoch and Seifert, 2012), which inherently leave fungal relative distributions with great uncertainties.

From a qualitative point of view, the communities of various areas in PW shared numerous taxa, overall in agreement with the findings of Bruez et al. (2014), although some tissue types were more variable than others (Figure 3). Also, considerable differences are evident from a quantitative point of view. High variability was observed among individual plants, as already noted in grapevines (Travadon et al., 2013), suggesting the need to increase the amount of biological replicates in future studies.

The graft union and the wood below the spurs host unique taxa or taxa that are found only in low percentages (RA < 0.1%; Table 1), or completely absent, in other parts of the plant. Supposedly, the root system (in this study *V. berlandieri* × *V. rupestris*), whose endosphere is influenced by the soil microbiome (Zarraonaindia et al., 2015) and by the rootstock used in the propagation process, harbor unique fungi that have a limited capacity of colonizing the stem of *V. vinifera*. Interestingly, *Fomitiporia* sp., a species that is well represented in all PW tissues (8.9% < RA < 22.9%), is nearly absent in the graft union (0.6%; Figure 4). This suggests that other species of *Vitis* may be more resistant than *V. vinifera* to *Fomitiporia* sp., in fact, artificial inoculations of *Fomitiporia punctata* in rootstock Kober 5BB (*V. berlandieri* × *Vitis riparia*) led to low re-isolation percentages of this pathogen (8% of inoculated plants; Sparapano et al., 2000a). We are not aware of any other paper in the literature that describes the presence of *Fomitiporia* sp. in the root system of naturally infected vines.

The spur tissue is located in proximity of pruning wounds, which are considered the main entry point of GTD-associated fungal pathogens (Bertsch et al., 2013). Through this woody tissue some colonizers can successfully spread throughout the cordon and trunk (e.g., *P. chlamydospora* or *Fomitiporia* sp.), while others may be restrained by the antagonistic interactions with the resident mycobiome and/or plant defenses (e.g., *Eutypa* spp., *Clonostachys rosea*, *Acremonium* sp.; Table 1).

The remaining tissues, namely the trunk, upper trunk and arm points, do not differ by any of the parameters measured in this study (e.g., alpha and beta diversity, MetacodeR; Figures 2, 3, 5, 6), where unique taxa among the most represented fungi are nearly absent (Table 1), and relative abundances differ primarily in the representation of *P. chlamydospora* (Figure 4).

The results of this study show that the wood mycobiome of grapevines may vary and, in order to have a representative understanding of the diversity and abundance of the fungal communities, multiple sampling areas are recommended. We propose four samples per plant. The first two are the graft union and the wood below one spur, where some taxa are uniquely represented and abundances vary considerably; the third is any point of the trunk or arm; and the forth is canes.

### Fungal Communities and Leaf Symptoms

The outcome of the statistical analyses of the mycobiome of both PW and canes highlights that fungal communities were not affected by the manifestation of leaf symptoms, or *vice versa* (Figures 7, 8 and Supplementary Figure S1). The manifestation of ‘tiger stripes’ leaf symptoms, always associated with an advanced stage of infection by tracheomycotic pathogens, is in large part cryptic due to its discontinuity and unidentified causal agents (Mugnai et al., 1999). Greenhouse and field trials often failed to reproduce such symptoms with artificial inoculations of *P. chlamydospora* and/or *P. minimum* (Zanzotto et al., 2007; Gramaje et al., 2010), except for one study in which esca-like leaf symptoms were replicated in a very small percentage of inoculated plants (Sparapano et al., 2001). Other microbial ecology studies of the wood from leaf-symptomatic and asymptomatic vines showed that there are no differences in the fungal community composition, finding similar abundances of wood pathogens (Hofstetter et al., 2012; Bruez et al., 2014). Nevertheless, tracheomycotic fungi are currently believed to be directly or indirectly responsible for leaf symptoms as they are frequently isolated from symptomatic wood (Surico et al., 2008). The two arguments supporting this hypothesis are that the translocation of (1) fungal toxins, (2) byproducts of the wood degradation or (3) a combination of both, via xylem sap, from PW to leaves, are responsible for the appearance of leaf symptoms (Mugnai et al., 1999). While other studies showed that some toxins or culture filtrates of tracheomycotic fungi could lead to leaf discoloration and necrosis, solid evidence of the replication of the ‘tiger stripes’ pattern, as well as symptoms fluctuation, is lacking (Evidente et al., 2000; Sparapano et al., 2000b); Abou-Mansour et al., 2004; Andolfi et al., 2011. No evidence is currently available in support of the second and third hypotheses, namely those involving the byproducts of wood degradation.

The results of the present study are, to some extent, in agreement with the findings of Hofstetter et al. (2012) and Bruez et al. (2014). In fact, the fungal communities present in the PW in proximity of leaves that exhibited symptoms were overall similar to those found in plants with healthy leaves (in symptomatic or asymptomatic plants). When considering trends, *P. chlamydospora* is more abundant in the wood near non-symptomatic leaves, while *Fomitiporia* sp. is the most represented taxa in the wood near symptomatic leaves. This observation suggests a higher wood decay activity, which likely leads to a greater presence of byproducts of wood degradation, therefore supporting the second hypothesis for leaf symptoms manifestation. Regardless, it does not explain the manifestation of leaf symptomatology in grapevines not infected by *Fomitiporia* sp. (Edwards et al., 2001).

The similarity in the community structure of symptomatic and non-symptomatic canes is an additional evidence in support of the current understanding that fungi present in canes are not directly linked with leaf symptomatology (Figures 7, 8 and Supplementary Figure S1).

Leaf symptoms manifestation remains cryptic, however, it is important to note that this study, as well as the one by Bruez et al. (2014), analyzed the microbial communities of PW several months after the plant had exhibited leaf symptoms. It is not known whether the pathogens abundance in the moment pre-/during/post-symptomatology varies. In fact, the study by Bruez et al. (2014) revealed that alterations in the wood mycobiome may occur in different seasons, therefore further research is needed in this direction. However, this may not apply to the canes, as leaf symptoms were still present, and manifested only 2 months before wood tissue sampling.

### Further Considerations

In this study, the mycobiome of the canes is composed principally by endophytes and saprobes, with the exception of pathogens *P. chlamydospora* and *Diaporthe* sp., whose presence did not induce the appearance of wood symptoms. Concerning the former, this observation suggests that this fungus may fall along a continuum ‘from mutualist to saprophyte or latent pathogen’ (Wearn et al., 2012), as also proposed by other authors (Rumbos and Rumbou, 2001; Hofstetter et al., 2012), and whose pathogenicity is triggered by external factors. Interestingly, also *Fomitiporia* sp. was often identified in woody tissue that did not present the typical white rot symptom. This fungus may live asymptomatically in wood when found in low abundances, and produce white rot when its presence increases considerably. Hypothetically, the wood cores showing this symptom (15% of the total) may be the same in which *Fomitiporia* sp. was present in a RA > 35% (17.5%).

The presence of *P. chlamydospora*, and that of other wood pathogens, in vineyards around the world might be currently blatantly underestimated due to the elusiveness of internal symptoms. The Almotivo vineyard (this study) presented an incidence of leaf symptoms manifestation of ca. 1%, for three consecutive years, nevertheless, 100% of the sampled plants were colonized by both *P. chlamydospora* (in PW and canes) and *Fomitiporia* sp. (in PW). It is already known that leaf symptoms are not a reliable parameter to assess the health status of a vineyard (Pollastro et al., 2000), although it is the one most frequently employed, along with the count of dead vines (Lecomte et al., 2018). It is of utmost importance to develop tools that allow vine growers to assess the real extent of infections in the wood, and apply appropriate control measures.

## Conclusion

The characterization of the grapevine wood mycobiome, using NGS, in a vineyard affected by esca proper is an important step that lays the foundations for future studies to compare microbial community structures of vineyards affected by esca or other GTD. Some parameters that may influence the mycobiome composition and which may be of interest to investigate upon are: the flora surrounding the vineyard, the climatic conditions and seasonality, geographical location and year. Moreover, useful comparisons can be made between cultivars and vineyard management strategies (e.g., conventional, organic, and biodynamic). Eventually, a meta-analysis of the mycobiome that takes into account several of these parameters may reveal a pattern that could elucidate some of the obscure points that still prevent a full understanding of the etiology of this disease complex.

## Author Contributions

GDF conceived the study, performed field sampling and wet-lab work, interpreted and discussed the results, and wrote the manuscript. AG performed the wet-lab work and bioinformatics analyses, interpreted the results, and wrote the manuscript. MA performed statistical analyses and data visualization. HO, LH, and RF supervised the study and reviewed the manuscript.

## Conflict of Interest Statement

The authors declare that the research was conducted in the absence of any commercial or financial relationships that could be construed as a potential conflict of interest.

## References

[B1] Abdelfattah A. Wisniewski M. Droby S. Schena L. (2016). Spatial and compositional variation in the fungal communities of organic and conventionally grown apple fruit at the consumer point-of-purchase. *Hortic. Res.* 3:16047. 10.1038/hortres.2016.47 27766161 PMC5051542

[B2] Abou-Mansour E. Couché E. Tabacchi R. (2004). Do fungal naphthalenones have a role in the development of esca symptoms? *Phytopathol. Mediterr.* 43 75–82.

[B3] Ahmed S. A. van de Sande W. W. J. Stevens D. A. Fahal A. van Diepeningen A. D. Menken S. B. J. (2014). Revision of agents of black-grain eumycetoma in the order Pleosporales. *Persoonia* 33 141–154. 10.3767/003158514X684744 25737597 PMC4312930

[B4] Andolfi A. Mugnai L. Luque J. Surico G. Cimmino A. Evidente A. (2011). Phytotoxins produced by fungi associated with grapevine trunk diseases. *Toxins* 3 1569–1605. 10.3390/toxins3121569 22295177 PMC3268457

[B5] Aurand J. M. (2017). “State of the vitiviniculture world market,” in *Proceedings of the 38th OIV World Congress of Vine and Wine*, Mainz.

[B6] Bakhshi M. Arzanlou M. (2017). Multigene phylogeny reveals a new species and novel records and hosts in the genus *Ramularia* from Iran. *Mycol. Prog.* 16 703–712. 10.1007/s11557-017-1308-y

[B7] Bertsch C. Ramírez-Suero M. Magnin-Robert M. Larignon P. Chong J. Abou-Mansour E. (2013). Grapevine trunk diseases: complex and still poorly understood. *Plant Pathol.* 62 243–265. 10.1111/j.1365-3059.2012.02674.x

[B8] Bruez E. Baumgartner K. Bastien S. Travadon R. Guérin-Dubrana L. Rey P. (2016). Various fungal communities colonise the functional wood tissues of old grapevines externally free from grapevine trunk disease symptoms. *Aust. J. Grape Wine Res.* 22 288–295. 10.1111/ajgw.12209

[B9] Bruez E. Vallance J. Gerbore J. Lecomte P. Da Costa J. P. Guerin-Dubrana L. (2014). Analyses of the temporal dynamics of fungal communities colonizing the healthy wood tissues of esca leaf-symptomatic and asymptomatic vines. *PLoS One* 9:e95928. 10.1371/journal.pone.0095928 24788412 PMC4006835

[B10] Callahan B. J. McMurdie P. J. Holmes S. P. (2017). Exact sequence variants should replace operational taxonomic units in marker-gene data analysis. *ISME J.* 11 2639–2643. 10.1038/ismej.2017.119 28731476 PMC5702726

[B11] Calzarano F. Di Marco S. (2018). Further evidence that calcium, magnesium and seaweed mixtures reduce grapevine leaf stripe symptoms and increase grape yield. *Phytopathol. Mediterr.* 57 459–471. 10.14601/Phytopathol_Mediterr-23636

[B12] Caporaso G. Kuczynski J. Stombaugh J. Bittinger K. Bushman F. D. Costello E. K. (2010). QIIME allows analysis of high-throughput community sequencing data. *Nat. Methods* 7 335–336. 10.1038/nmeth0510-335 20383131 PMC3156573

[B13] Casieri L. Hofstetter V. Viret O. Gindro K. (2009). Fungal communities living in the wood of different cultivars of young *Vitis vinifera* plants. *Phytopathol. Mediterr.* 48 73–83.

[B14] Connell L. Staudigel H. (2013). Fungal diversity in a dark oligotrophic volcanic ecosystem (DOVE) on Mount Erebus, Antarctica. *Biology* 2 798–809. 10.3390/biology2020798 24832809 PMC3960884

[B15] Csardi G. Nepusz T. (2006). The igraph software package for complex network research. *Int. J. Complex Syst.* 1695 1–9.

[B16] Darrieutort G. Pascal L. (2007). Evaluation of a trunk injection technique to control grapevine trunk diseases. *Phytopathol. Mediterr.* 46 50–57. 10.14601/-1853

[B17] Dissanayake A. J. Purahong W. Wubet T. Hyde K. D. Zhang W. Xu H. (2018). Direct comparison of culture-dependent and culture-independent molecular approaches reveal the diversity of fungal endophytic communities in stems of grapevine (*Vitis vinifera*). *Fungal Divers.* 90 85–107. 10.1007/s13225-018-0399-3

[B18] Dowle M. Srinivasan A. (2017). *Data Table: Extension of Data Frame.* Available at: https://cran.r-project.org/web/packages/data.table/index.html (accessed January 8, 2019).

[B19] Dray S. Blanchet G. Borcard D. Clappe S. Guenard G. Jombart T. (2018). *A Despatial: Multivariate Multiscale Spatial Analysis. R Package Version* 0.1-1. Available at: https://cran.r-project.org/web/packages/adespatial/index.html (accessed January 8, 2019).

[B20] Edwards J. Marchi G. Pascoe I. G. (2001). Young esca in Australia. *Phytopathol. Mediterr.* 40 303–310. 10.14601/Phytopathol

[B21] Edwards J. Pascoe I. G. (2004). Occurrence of *Phaeomoniella chlamydospora* and *Phaeoacremonium aleophilum* associated with petri disease and esca in Australian grapevines. *Aust. Plant Pathol.* 33 273–279. 10.1071/AP04016

[B22] Eskalen A. Feliciano A. Gubler W. (2007). Susceptibility of grapevine pruning wounds and symptom development in response to infection by *Phaeoacremonium aleophilum* and *Phaeomoniella chlamydospora*. *Plant Dis.* 91 1100–1104. 10.1094/Pdis-91-9-1100 30780648

[B23] Evidente A. Sparapano L. Andolfi A. Bruno G. (2000). Two naphthalenone pentaketides from liquid cultures of *Phaeoacremonium aleophilum*, a fungus associated with esca of grapevine. *Phytopathol. Mediterr.* 39 162–168. 10.14601/Phytopathol_Mediterr-1559

[B24] Feld L. Nielsen T. K. Hansen L. H. Aamand J. Albers C. N. (2015). Establishment of bacterial herbicide degraders in a rapid sand filter for bioremediation of phenoxypropionate-polluted groundwater. *Appl. Environ. Microbiol.* 82 878–887. 10.1128/AEM.02600-15 26590282 PMC4725289

[B25] Fontaine F. Gramaje D. Armengol J. Smart R. Nagy Z. A. Borgo M. (2016). *Grapevine Trunk Diseases. A Review.* Paris: OIV publications.

[B26] Foster Z. Sharpton T. Grünwald N. (2017). Metacoder: an R package for visualization and manipulation of community taxonomic diversity data. *PLoS Comput. Biol.* 13:1005404. 10.1371/journal.pcbi.1005404 28222096 PMC5340466

[B27] Ganter P. F. Starmer W. T. Lachance M. A. Phaff H. J. (1986). Yeast communities from host plants and associated *Drosophila* in southern arizona: new isolations and analysis of the relative importance of hosts and vectors on comunity composition. *Oecologia* 70 386–392. 10.1007/BF00379501 28311925

[B28] Gaylarde C. Ogawa A. Beech I. Kowalski M. Baptista-Neto J. A. (2017). Analysis of dark crusts on the church of nossa senhora do carmo in rio de janeiro, Brazil, using chemical, microscope and metabarcoding microbial identification techniques. *Int. Biodeterior. Biodegradation* 117 60–67. 10.1016/j.ibiod.2016.11.028

[B29] Gobbi A. Santini R. Filippi E. Ellegaard-Jensen L. Jacobsen C. S. Hansen L. H. (2019). Quantitative and qualitative evaluation of the impact of the G2 enhancer, bead sizes and lysing tubes on the bacterial community composition during DNA extraction from recalcitrant soil core samples based on community sequencing and qPCR. *PLoS One* 14:e0200979. 10.1371/journal.pone.0200979 30973938 PMC6459482

[B30] González A. E. Martínez A. T. Almendros G. Grinbergs J. (1989). A study of yeasts during the delignification and fungal transformation of wood into cattle feed in Chilean rain forest. *Antonie Van Leeuwenhoek* 55 221–236. 10.1007/BF00393851 2757365

[B31] González V. Sánchez-Torres P. Hinarejos R. Tuset J. J. (2009). *Inonotus hispidus* fruiting bodies on grapevines with esca symptoms in mediterranean areas of Spain. *J. Plant Pathol.* 91 465–468.

[B32] González V. Tello M. L. (2011). The endophytic mycota associated with *Vitis vinifera* in central Spain. *Fungal Divers.* 47 29–42. 10.1007/s13225-010-0073-x

[B33] Gramaje D. García-Jiménez J. Armengol J. (2010). Field evaluation of grapevine rootstocks inoculated with fungi associated with petri disease and esca. *Am. J. Enol. Vitic.* 61 512–520. 10.5344/ajev.2010.10021

[B34] Gramaje D. Urbez-Torres J. R. Sosnowski M. R. (2018). Managing grapevine trunk diseases with respect to etiology and epidemiology: current strategies and future prospects. *Plant Dis.* 102 12–39. 10.1094/PDIS-04-17-0512-FE 30673457

[B35] Halleen F. Crous P. W. Petrini O. (2003). Fungi associated with healthy grapevine cuttings in nurseries, with special reference to pathogens involved in the decline of young vines. *Aust. Plant Pathol.* 32 47–52. 10.1071/AP02062

[B36] Haňáčková Z. Havrdová L. Černý K. Zahradník D. Koukol O. (2017). Fungal endophytes in ash shoots – diversity and inhibition of *Hymenoscyphus fraxineus*. *Balt. For.* 23 89–106.

[B37] Haynes J. D. (2016). The developmental morphology of *Anthostoma gastrinum*. *Mycologia* 61 518–525. 10.1080/00275514.1969.12018765

[B38] Hofstetter V. Buyck B. Croll D. Viret O. Couloux A. Gindro K. (2012). What if esca disease of grapevine were not a fungal disease? *Fungal Divers.* 54 51–67. 10.1007/s13225-012-0171-z

[B39] Jaklitsch W. M. Fournier J. Rogers J. D. Voglmayr H. (2014). Phylogenetic and taxonomic revision of *Lopadostoma*. *Persoonia* 32 52–82. 10.3767/003158514X679272 25264383 PMC4150080

[B40] Jara C. Laurie V. F. Mas A. Romero J. (2016). Microbial terroir in chilean valleys: diversity of non-conventional yeast. *Front. Microbiol.* 7:663. 10.3389/fmicb.2016.00663 27242693 PMC4868835

[B41] Jayawardena R. S. Purahong W. Zhang W. Wubet T. Li X. Liu M. (2018). Biodiversity of fungi on *Vitis vinifera* L. revealed by traditional and high-resolution culture-independent approaches. *Fungal Divers.* 90 1–84. 10.1007/s13225-018-0398-4

[B42] Kane D. F. Tam W. Y. Jones E. B. G. (2002). Fungi colonising and sporulating on submerged wood in the river severn, UK. *Fungal Divers.* 10 45–55.

[B43] Kolařík M. Spakowicz D. J. Gazis R. Shaw J. Kubátová A. Nováková A. (2017). *Biatriospora* (Ascomycota: Pleosporales) is an ecologically diverse genus including facultative marine fungi and endophytes with biotechnological potential. *Plant Syst. Evol.* 303 35–50. 10.1007/s00606-016-1350-2

[B44] Kovalchuk A. Mukrimin M. Zeng Z. Raffaello T. Liu M. Kasanen R. (2018). Mycobiome analysis of asymptomatic and symptomatic Norway spruce trees naturally infected by the conifer pathogens *Heterobasidion* spp. *Environ. Microbiol. Rep.* 10 532–541. 10.1111/1758-2229.12654 29727054

[B45] Larignon P. Fontaine F. Farine S. Clément C. Bertsch C. (2009). Esca et black dead arm?: deux acteurs majeurs des maladies du bois chez la vigne. *C. R. Biol.* 332 765–783. 10.1016/j.crvi.2009.05.005 19748452

[B46] Lecomte P. Diarra B. Carbonneau A. Rey P. Chevrier C. (2018). Esca of grapevine and training practices in France : results of a 10-year survey. *Phytopathol. Mediterr.* 57 472–487. 10.14601/Phytopathol_Mediterr-22025

[B47] McMurdie P. J. Holmes S. (2013). Phyloseq: an R package for reproducible interactive analysis and graphics of microbiome census data. *PLoS One* 8:e61217. 10.1371/journal.pone.0061217 23630581 PMC3632530

[B48] McMurdie P. J. Paulson J. N. (2018). *Biomformat: an Interface Package for the BIOM File Format.* Available at: https://github.com/joey711/biomformat (accessed January 8, 2019).

[B49] Mehrabi M. Hemmati R. (2015). Two new records of *Lopadostoma* for mycobiota of Iran. *Mycol. Iran.* 2 59–64.

[B50] Middelhoven W. J. (2003). The yeast flora of the coast redwood, *Sequoia sempervirens*. *Folia Microbiol.* 48 361–362. 10.1007/BF02931367 12879747

[B51] Miguel P. S. B. Delvaux J. C. de Oliveira M. N. V. Moreira B. C. Borges A. C. Totola M. R. (2017). Diversity and distribution of the endophytic fungal community in eucalyptus leaves. *Afr. J. Microbiol. Res.* 11 92–105. 10.5897/AJMR2016.8353

[B52] Mondello V. Songy A. Battiston E. Pinto C. Coppin C. Trotel-Aziz P. (2017). Grapevine trunk diseases: a review of fifteen years of trials for their control with chemicals and biocontrol agents. *Plant Dis.* 102:7. 10.1094/PDIS-08-17-1181-FE 30673583

[B53] Morgan H. H. du Toit M. Setati M. E. (2017). The grapevine and wine microbiome: insights from high-throughput amplicon sequencing. *Front. Microbiol.* 8:820. 10.3389/fmicb.2017.00820 28553266 PMC5425579

[B54] Mostert L. Halleen F. Fourie P. Crous P. W. (2006). A review of *Phaeo-acremonium* species involved in petri disease and esca of grapevines. *Phytopathol. Mediterr.* 45(Suppl. 1), 12–29.

[B55] Mugnai L. Graniti A. Surico G. (1999). Esca (black measles) and brown wood-streaking: two old and elusive diseases of grapevines. *Plant Dis.* 83 404–418. 10.1094/PDIS.1999.83.5.404 30845530

[B56] Nilsson R. H. Taylor A. F. S. Bates S. T. Thomas D. Bengtsson-Palme J. Callaghan T. M. (2013). Towards a unified paradigm for sequence-based identification of fungi. *Mol. Ecol.* 22 5271–5277. 10.1111/mec.12481 24112409

[B57] Oksanen J. Kindt R. Legendre P. O’Hara B. Stevens M. H. H. Oksanen M. J. (2007). The vegan package. *Commun. Ecol. Package* 10 631–637.

[B58] Pancher M. Ceol M. Corneo P. E. Longa C. M. O. Yousaf S. Pertot I. (2012). Fungal endophytic communities in grapevines (*Vitis vinifera* L.) respond to crop management. *Appl. Environ. Microbiol.* 78 4308–4317. 10.1128/AEM.07655-11 22492448 PMC3370515

[B59] Peay K. Kennedy P. G. Talbot J. (2016). Dimensions of biodiverisity in the Earth mycobiome. *Nature* 14 434–447. 10.1038/nrmicro.2016.59 27296482

[B60] Phaff H. J. Vaughan-Martini A. Starmer W. T. (1998). *Debaryomyces* prosopidis sp. nov., a yeast from exudates of mesquite trees. Int. J. Syst. Bacteriol. 48 1419–1424. 10.1099/00207713-48-4-1419

[B61] Pinto C. Pinho D. Sousa S. Pinheiro M. Egas C. Gomes A. C. (2014). Unravelling the diversity of grapevine microbiome. *PLoS One* 9:e85622. 10.1371/journal.pone.0085622 24454903 PMC3894198

[B62] Pollastro S. Dongiovanni C. Abbatecola A. Faretra F. (2000). Observations on the fungi associated with esca and on spatial distribution of esca-symptomatic plants in Apulian (Italy) vineyards. *Phytopathol. Mediterr.* 39 206–210.

[B63] Qi F. Jing T. Zhan Y. (2012). Characterization of endophytic fungi from *Acer ginnala* Maxim in an artificial plantation: media effect and tissue-dependent variation. *PLoS One* 7:e46785. 10.1371/journal.pone.0046785 23056451 PMC3466316

[B64] Rumbos I. Rumbou A. (2001). Fungi associated with esca and young grapevine decline in Greece. *Phytopathol. Mediterr.* 40 330–335. 10.1002/pssa.2210290216

[B65] Schoch C. L. Seifert K. A. (2012). Nuclear ribosomal internal transcribed spacer (ITS) region as a universal DNA barcode marker for fungi. *Proc. Natl. Acad. Sci. U.S.A.* 109:E1812 10.1073/pnas.1207508109 PMC3341068 22454494

[B66] Singh D. K. Sharma V. K. Kumar J. Mishra A. Verma S. K. Sieber T. N. (2017). Diversity of endophytic mycobiota of tropical tree *Tectona grandis* Linn.f.: spatiotemporal and tissue type effects. *Sci. Rep.* 7 1–14. 10.1038/s41598-017-03933-0 28623306 PMC5473821

[B67] Singh P. Santoni S. This P. Péros J.-P. (2018). Genotype-environment interaction shapes the microbial assemblage in grapevine’s phyllosphere and carposphere: an NGS approach. *Microorganisms* 6:96. 10.3390/microorganisms6040096 30248973 PMC6313654

[B68] Sparapano L. Bruno G. Ciccarone C. Graniti A. (2000a). Infection of grapevines by some fungi associated with esca. I. *Fomitiporia punctata* as a wood-rot inducer. *Phytopathol. Mediterr.* 39 46–52. 10.14601/PHYTOPATHOL_MEDITERR-1542

[B69] Sparapano L. Bruno G. Graniti A. (2000b). Effects on plants of metabolites produced in culture by *Phaeoacremonium chlamydosporum*, *P. aleophilum* and *Fomitiporia punctata*. *Phytopathol. Mediterr.* 39 169–177. 10.14601/phytopathol_mediterr-1535

[B70] Sparapano L. Bruno G. Graniti A. (2001). Three-year observation of grapevines cross-inoculated with esca-associated fungi. *Phytopathol. Mediterr.* 40 376–386.

[B71] Suetrong S. Hyde K. D. Zhang Y. Bahkali A. H. Jones G. (2011). Trematosphaeriaceae fam. nov. *(Dothideomycetes, Ascomycota)*. *Cryptogam. Mycol.* 32 343–358. 10.7872/crym.v32.iss4.2011.343

[B72] Surico G. (2009). Towards a redefinition of the diseases within the esca complex of grapevine. *Phytopathol. Mediterr.* 48 5–10.

[B73] Surico G. Mugnai L. Marchi G. (2008). “The esca disease complex,” in *Integrated Management of Diseases Caused by Fungi, Phytoplasma and Bacteria*, eds Ciancio A. Mukerji K. G. (Berlin: Springer), 119–136. 10.1007/978-1-4020-8571-0_6

[B74] Thambugala K. M. Hyde K. D. Tanaka K. Tian Q. Wanasinghe D. N. Ariyawansa H. A. (2015). Towards a natural classification and backbone tree for Lophiostomataceae, Floricolaceae, and Amorosiaceae fam. nov. *Fungal Divers.* 74 199–266. 10.1007/s13225-015-0348-3

[B75] Travadon R. Lecomte P. Diarra B. Lawrence D. P. Renault D. Ojeda H. (2016). Grapevine pruning systems and cultivars influence the diversity of wood-colonizing fungi. *Fungal Ecol.* 24 82–93. 10.1016/j.funeco.2016.09.003

[B76] Travadon R. Rolshausen P. E. Gubler W. D. Cadle-Davidson L. Baumgartner K. (2013). Susceptibility of cultivated and wild Vitis spp. to wood infection by fungal trunk pathogens. *Plant Dis.* 97 1529–1536. 10.1094/PDIS-05-13-0525-RE 30716856

[B77] Varela C. Borneman A. R. (2017). Yeasts found in vineyards and wineries. *Yeast* 34 111–128. 10.1002/yea.3219 27813152

[B78] Wearn J. A. Sutton B. C. Morley N. J. Gange A. C. (2012). Species and organ specificity of fungal endophytes in herbaceous grassland plants. *J. Ecol.* 100 1085–1092. 10.1111/j.1365-2745.2012.01997.x

[B79] Wickham H. (2016). *Ggplot2: Elegant Graphics for Data Analysis.* Berlin: Springer.

[B80] Xing X. K. Chen J. Xu M. J. Lin W. H. Guo S. X. (2011). Fungal endophytes associated with *Sonneratia* (Sonneratiaceae) mangrove plants on the south coast of China. *For. Pathol.* 41 334–340. 10.1111/j.1439-0329.2010.00683.x

[B81] Zanzotto A. Autiero F. Bellotto D. Dal Cortivo G. Lucchetta G. Borgo M. (2007). Occurrence of *Phaeoacremonium* spp. and *Phaeomoniella chlamydospora* in grape propagation materials and young grapevines. *Eur. J. Plant Pathol.* 119 183–192. 10.1007/s10658-007-9160-6

[B82] Zarraonaindia I. Owens S. M. Weisenhorn P. West K. Hampton-marcell J. Lax S. (2015). The soil microbiome influences grapevine-associated microbiota. *mBio* 6 1–10. 10.1128/mBio.02527-14.Editor 25805735 PMC4453523

[B83] Zhou Y. H. Zhang M. Zhu R.-X. Zhang J.-Z. Xie F. Li X.-B. (2016). Heptaketides from an endolichenic fungus *Biatriospora* sp. and their antifungal activity. *J. Nat. Prod.* 79 2149–2157. 10.1021/acs.jnatprod.5b00998 27556953

