# Supplementary material for: Characterization of the Wood Mycobiome of Vitis vinifera in a Vineyard Affected by Esca. Spatial Distribution of Fungal Communities and Their Putative Relation With Leaf Symptoms
Source: Front Plant Sci. 2019 Jul 12;10:910. doi: 10.3389/fpls.2019.00910 (PMC6640213; doi:10.3389/fpls.2019.00910)
Supplement: Supplementary file 4 [file Data_Sheet_4.docx]

**Supplementary material**

Supplementary Table 1. Classification of the woody plants encountered in the proximity of the vineyard used in the present study.

| **Phylum** | **Class** | **Order** | **Family** | **Genus** | **Species** |
| --- | --- | --- | --- | --- | --- |
| Magnoliophyta | Liliopsida | Liliales | Agavaceae | *Agave* | *A. americana* |
| - | - | - | Asphodelaceae | *Aloe* | *A. arborescens* |
| - | Magnoliopsida | Apiales | Pittosporaceae | *Pittosporum* | *P. undulatum* |
| - | - | Caryophyllales | Cactaceae | *Opuntia* | *O. ficus-indica* ***** |
| - | - | - | - | *-* | *O. stricta* |
| - | - | Fabales | Fabaceae | *Ceratonia* | *C. siliqua* |
| - | - | - | - | *Cercis* | *C. siliquastrum* |
| - | - | Fagales | Fagaceae | *Quercus* | *Q. rotundifolia* |
| - | - | Lamiales | Oleaceae | *Fraxinus* | *F. angustifolia* |
| - | - | - | - | *-* | *F. australis* ***** |
| - | - | - | - | *Olea* | *O. silvestrys* ***** |
| - | - | - | - | *Phillyrea* | *P. latifolia* ***** |
| - | - | - | - | *-* | *P. media* ***** |
| - | - | Myrtales | Myrtaceae | *Eucalyptus* | *E. camaldulensis* |
| - | - | - | - | *-* | *E. globulus* |
| - | - | Proteales | Platanaceae | *Platanus* | *P. occidentalis* |
| - | - | Rhamnales | Rhamnaceae | *Rhamnus* | *R. alaternus* |
| - | - | Rosales | Rosaceae | *Eriobotrya* | *E. japonica* |
| - | - | - | - | *Prunus* | *P. dulcis* |
| - | - | - | - | *-* | *P. persica* |
| - | - | - | Moraceae | *Maclura* | *M. pomifera* |
| - | - | Urticales | Ulmaceae | *Celtis* | *C. australis* ***** |
| Pinophyta | Pinopsida | Pinales | Cupressaceae | *Cupressus* | *C. sempervirens* |
| - | - | - | Pinaceae | *Pinus* | *P. pinea* |

(*) denotes the most abundant species in the proximities of the Almotivo vineyard.

Supplementary Table 2. List of taxa identified to genus or species level present in the dataset including all sampling points for all tissue types –objective (1)-. Three groups are created to separate taxa present in a relative abundance (RA) greater than 0.1%, included between 0.1% and 0.01%, and lower than 0.01%. The taxa with a RA < 0.1% are considered rare taxa. Taxa followed by (*) are part of the core mycobiome – shared by perennial wood and canes –, taxa followed by (†) are unique to canes, taxa not followed by any symbol are unique to perennial wood.

| **RA > 0.1% (n= 50)** | **0.01% < RA < 0.1% (n= 146)** (*continues*) | *Wallemia muriae* * |
| --- | --- | --- |
| *Acremonium* sp. | *Cyphellophora* sp. | *Xanthoria* sp. |
| *Acremonium alternatum* * | *Cystobasidium pinicola* | *Xylodon sambuci* |
| *Alternaria* sp. * | *Cystobasidium* sp. | *Zymoseptoria* sp. |
| *Angustimassarina acerina* | *Cystofilobasidium capitatum* | **RA < 0.01% (n= 93)** |
| *Anthostoma gastrinum* | *Cystofilobasidium macerans* | *Acremonium fusidioides* |
| *Aureobasidium pullulans* * | *Devriesia pseudoamericana* * | *Agaricus blazei* |
| *Biatriospora mackinnonii* | *Devriesia* sp. | *Alternaria brassicae* |
| *Candida friedrichii* * | *Dioszegia hungarica* | *Alternaria eureka* |
| *Capronia coronata* | *Erythrobasidium hasegawianum* | *Alternaria metachromatica* |
| *Cladosporium* sp. * | *Eucasphaeria capensis* * | *Apiotrichum domesticum* |
| *Clonostachys rosea* | *Eutypa flavovirens* | *Arthrinium* sp. |
| *Colletotrichum* sp. * | *Eutypella citricola* | *Arthrobotrys superba* |
| *Cryptococcus* sp. * | *Exidia japonica* | *Ascochyta medicaginicola* |
| *Cryptococcus heimaeyensis* | *Exobasidium* sp. † | *Aspergillus amstelodami* |
| *Cryptococcus victoriae* * | *Exophiala oligosperma* | *Aspergillus ochraceus* |
| *Debaryomyces* sp. * | *Fellomyces* sp. | *Beauveria bassiana* |
| *Debaryomyces prosopidis* * | *Fomitiporella* sp. | *Bjerkandera adusta* |
| *Diaporthe* sp. * | *Funneliformis geosporum* | *Candida etchellsii* |
| *Eutypa lata* | *Fusarium poae* | *Candida mycetangii* |
| *Eutypa leptoplaca* | *Fusarium solani* | *Candida zeylanoides* |
| *Exophiala* sp. | *Ganoderma australe* | *Capronia pulcherrima* |
| *Exophiala xenobiotica* | *Ganoderma lucidum* † | *Catenulostroma hermanusense* |
| *Filobasidium globisporum* | *Ganoderma resinaceum* | *Cladosporium salinae* |
| *Filobasidium magnum* * | *Gibellulopsis chrysanthemi* | *Colacogloea* sp. |
| *Fomitiporia* sp. | *Holtermanniella takashimae* | *Cryptococcus aureus* |
| *Fomitiporia mediterranea* | *Hyphodontia alutaria* | *Cryptosphaeria subcutanea* |
| **RA > 0.1% (n= 50)** (*continues*) | **0.01% < RA < 0.1% (n= 146)** (*continues*) | **RA < 0.01% (n= 93)** (*continues*) |
| *Fusarium* sp. * | *Hyphodontia radula* * | *Cryptovalsa ampelina* |
| *Glomerella acutata* * | *Knufia epidermidis* | *Curvularia* sp. |
| *Guehomyces pullulans* | *Knufia perforans* | *Curvularia tsudae* |
| *Inonotus hispidus* | *Laetiporus sulphureus* | *Cyphellophora europaea* |
| *Lopadostoma meridionale* | *Leprocaulon* sp. † | *Cyphellophora reptans* |
| *Lopadostoma quercicola* | *Meira nashicola* * | *Cystofilobasidium infirmominiatum* |
| *Lophiostoma cynaroidis* | *Microdiplodia* sp. | *Cytospora* sp. |
| *Lophiostoma* sp. | *Minimedusa polyspora* | *Debaryomyces mycophilus* |
| *Lophiotrema rubi* | *Mortierella* sp. | *Dioszegia zsoltii var. yunnanensis* |
| *Malassezia globosa* * | *Mortierella minutissima* | *Diplodia pseudoseriata* |
| *Malassezia restricta* * | *Mrakia* sp. | *Engyodontium album* |
| *Massarina* sp. | *Mucor* sp. | *Epicoccum pimprinum* † |
| *Meyerozyma guilliermondii* * | *Mycena metata* | *Erysiphe necator* |
| *Mycosphaerella tassiana* * | *Naganishia albidosimilis* | *Erythrobasidium elongatum* |
| *Penicillium* sp. * | *Neodevriesia capensis* | *Erythrobasidium* sp. |
| *Peniophora* sp. * | *Neoerysiphe galeopsidis* | *Exophiala bergeri* |
| *Phaeomoniella chlamydospora* * | *Neofusicoccum* sp. * | *Fellomyces penicillatus* |
| *Psathyrella* sp. | *Neofusicoccum parvum* | *Fellomyces polyborus* |
| *Ramularia* sp. * | *Neofusicoccum australe* * | *Filobasidium wieringae* |
| *Rhinocladiella* sp. | *Neonectria* sp. | *Fuscoporia ferruginosa* |
| *Rhodotorula mucilaginosa* * | *Occultifur* sp. | *Gymnopus barbipes* |
| *Sporidiobolus* sp. | *Orbilia* sp. | *Hannaella* sp. |
| *Trematosphaeria pertusa* | *Papiliotrema flavescens* | *Hanseniaspora* sp. † |
| *Vishniacozyma carnescens* | *Paraconiothyrium* sp. * | *Heterobasidion irregulare* † |
|  | *Paraphaeosphaeria parmeliae* | *Hyphoderma nudicephalum* |
| **0.01% < RA < 0.1% (n= 146)** | *Penicillium citreonigrum* | *Ilyonectria liriodendri* |
| *Absidia* sp. | *Peniophorella pubera* * | *Itersonilia pannonica* † |
| *Acremonium brunnescens* | *Petriella* sp. | *Knufia tsunedae* |
| *Annulohypoxylon* sp. | *Phacidiella eucalypti* | *Kondoa aeria* |
| *Apiotrichum* sp. | *Phaeomoniella* sp. | *Kurtzmanomyces* sp. |
| *Articulospora* sp. | *Phallus impudicus* | *Lachancea thermotolerans* |
| *Ascobolus* sp. | *Phanerochaete* sp. | *Lycoperdon ericaeum* |
| *Aspergillus* sp. | *Phialemoniopsis ocularis* | *Magnaporthe grisea* |
| *Aspergillus conicus* | *Phialophora cyclaminis* | *Malassezia* sp. * |
| *Aspergillus penicillioides* * | *Phialophora verrucosa* | *Malassezia sympodialis* |
| *Aspergillus proliferans* | *Physcia* sp. | *Mariannaea superimposita* |
| **0.01% < RA < 0.1% (n= 146)** (*continues*) | **0.01% < RA < 0.1% (n= 146)** (*continues*) | **RA < 0.01% (n= 93)** (*continues*) |
| *Bensingtonia* sp. | *Pleospora fallens* | *Mollisia cinerea* |
| *Bipolaris* sp. | *Podospora* sp. | *Monographella cucumerina* |
| *Blumeria graminis* | *Pyrenochaeta* sp. | *Monographella nivalis* |
| *Boeremia exigua* | *Pyrenochaeta keratinophila* | *Mortierella alpina* |
| *Buckleyzyma* sp. | *Pyrenochaeta unguis-hominis* | *Mucor hiemalis* |
| *Caloplaca obscurella* | *Pyrenophora tritici-repentis* | *Mucor saturninus* |
| *Candida* sp. | *Ramicandelaber* sp. | *Naganishia albida* |
| *Candida palmioleophila* | *Ramichloridium cucurbitae* | *Naganishia randhawae* |
| *Candida parapsilosis* | *Rhizomucor pusillus* | *Neodevriesia simplex* |
| *Candida sake* | *Rhizopus microsp.orus* | *Occultifur externus* |
| *Candida tropicalis* * | *Rhodotorula* sp. | *Papiliotrema pseudoalba* |
| *Candida orthopsilosis* | *Rhodotorula graminis* | *Paraphoma fimeti* |
| *Capnodium* sp. | *Rhodotorula nothofagi* | *Parasola conopilus* |
| *Capronia* sp. | *Rhodotorula diobovata* | *Periconia pseudobyssoides* |
| *Cenococcum* sp. | *Saccharomyces cerevisiae* | *Periconia* sp. |
| *Ceratobasidium cornigerum* | *Sakaguchia dacryoidea* | *Phialemoniopsis curvata* |
| *Ceratobasidium* sp. | *Sarcoporia* sp. | *Phlebia acerina* |
| *Circinotrichum maculiforme* * | *Sarocladium subulatum* * | *Phlebiopsis gigantea* † |
| *Citeromyces matritensis* | *Scheffersomyces spartinae* | *Pseudocercospora* sp. |
| *Cladophialophora chaetospira* | *Schizophyllum commune* | *Ramularia stellenboschensis* † |
| *Cladophialophora* sp. | *Sclerostagonospora* sp. | *Rhodotorula ingeniosa* |
| *Cladosporium delicatulum* * | *Scytalidium* sp. | *Rhodotorula terpenoidalis* |
| *Cladosporium fusiforme* | *Sistotremastrum* sp. * | *Rhodotorula toruloides* |
| *Cladosporium sphaerospermum* * | *Solicoccozyma terrea* | *Sclerostagonospora cycadis* |
| *Clathrus ruber* | *Sporobolomyces oryzicola* | *Scopuloides rimosa* |
| *Clitopilus* sp. | *Stemphylium* sp. | *Setophaeosphaeria badalingensis* |
| *Colletotrichum acerbum* * | *Tetracladium* sp. | *Sistotremastrum guttuliferum* |
| *Colletotrichum gloeosporioides* * | *Torulaspora delbrueckii* | *Sterigmatomyces halophilus* |
| *Coprinellus micaceus* | *Trametes hirsuta* | *Taphrina deformans* |
| *Cryptococcus aerius* | *Trichaptum abietinum* | *Trametes versicolor* |
| *Cryptococcus frias* | *Trichoderma* sp. * | *Trichosporon asahii* |
| *Cryptococcus uniguttulatus* | *Trichoderma harzianum* † | *Uncispora sinensis* |
| *Cutaneotrichosporon* sp. | *Veronaea compacta* | *Valsaria insitiva* |
| *Cutaneotrichosporon cyanovorans* | *Verticillium* sp. * | *Verrucocladosporium dirinae* |
| *Cyberlindnera jadinii* | *Wallemia* sp. | *Yamadazyma triangularis* |

Supplementary Table 3. One-way ANOVA with post-hoc Tukye’s HSD of woody tissue types to assess differences in the Alpha diversity (Shannon index, Pielou’s evenness) of fungal communities. For each row, column (A) is significantly different from column (B).

| **Index** | **A** | **B** | **P value** |
| --- | --- | --- | --- |
| **Shannon** | Canes | Spur_1 | 0.023 |
|  | Canes | Spur_2 | 0.027 |
|  | Upper_trunk | Spur_1 | 0.024 |
|  | Upper_trunk | Spur_2 | 0.028 |
| **Pielou's evenness** | Canes | Spur_1 | 0.005 |
|  | Canes | Spur_2 | 0.016 |
|  | Graft_Union | Spur_1 | 0.010 |
|  | Graft_Union | Spur_2 | 0.029 |
|  | Trunk | Spur_1 | 0.034 |
|  | Upper_trunk | Spur_1 | 0.006 |
|  | Upper_trunk | Spur_2 | 0.020 |


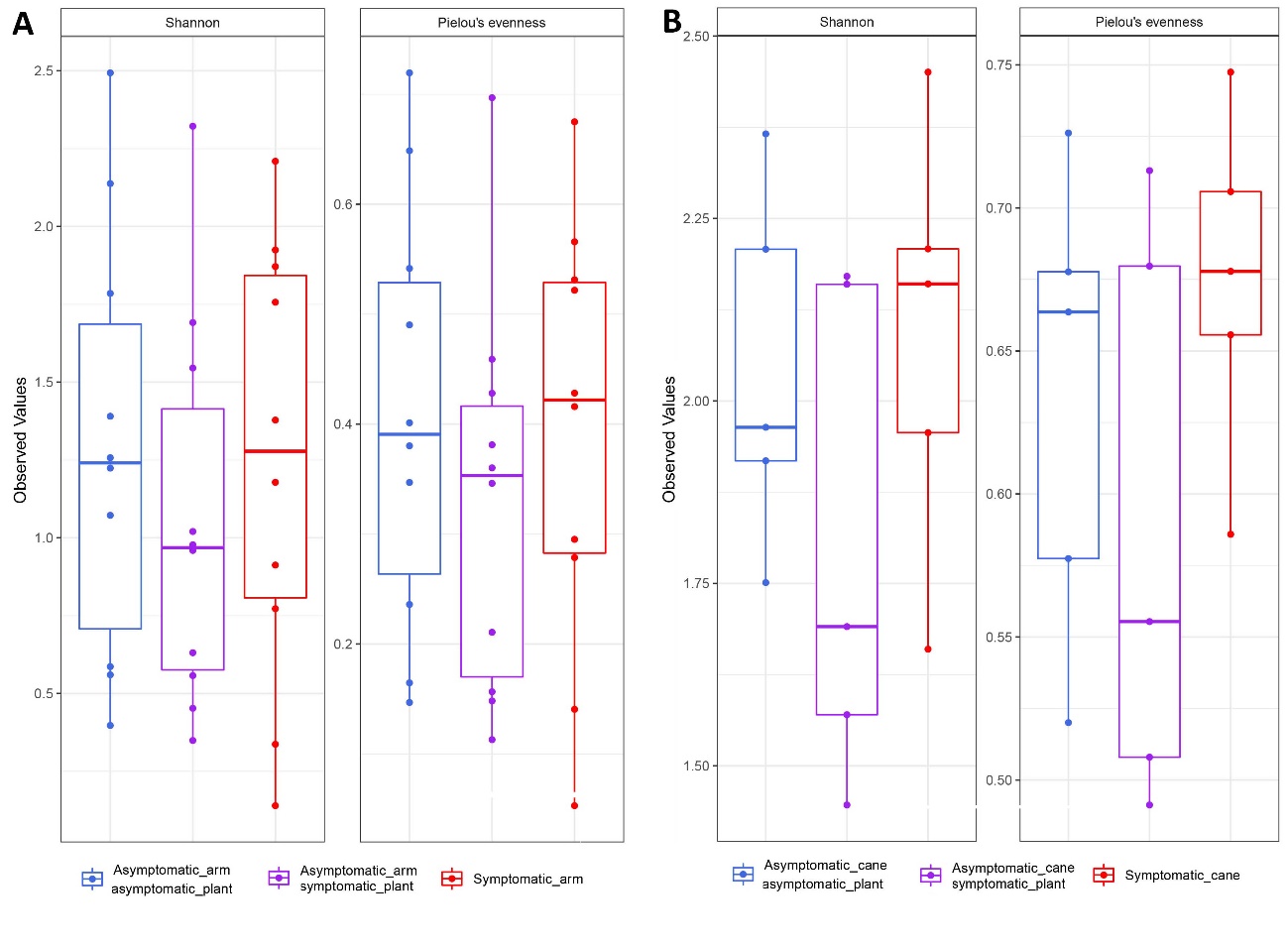


Figure S1. Box plots of diversity indexes (Shannon, Pielou’s evenness) of the fungal communities present in (A) perennial wood or (B) annual wood. (A) Communities found in the wood in proximity of symptomatic canopy (‘Symptomatic_arm’) or of asymptomatic canopy, either in symptomatic plants (‘Asymptomatic _arm symptomatic_plant’) or in asymptomatic plants (‘Asymptomatic_arm asymptomatic_plant’). (B) Communities found in canes with manifested foliar symptoms (‘Symptomatic_cane’) or asymptomatic, but coming from symptomatic plants (‘Asymptomatic_cane symptomatic_plant’) or asymptomatic plants (‘Asymptomatic_cane asymptomatic_plant’).
